# Performance Optimization of Nonorthogonal MFSK for Symbol-by-Symbol Coherent Detection

**DOI:** 10.3390/s26082293

**Published:** 2026-04-08

**Authors:** Luca Rugini

**Affiliations:** Dipartimento di Ingegneria, Università degli Studi di Perugia, 06215 Perugia, Italy; luca.rugini@unipg.it

**Keywords:** coherent detection, crosscorrelation, MFSK, non-equispaced frequencies, symbol error probability

## Abstract

M-ary frequency-shift keying (MFSK) is employed for several applications, including Internet-of-Things (IoT) and sensor-based communications. Previous studies have demonstrated that coherent detection of well-designed nonorthogonal MFSK signals outperforms orthogonal MFSK. This paper optimizes the error performance of nonorthogonal MFSK signals when the receiver uses a simple coherent detector on a symbol-by-symbol basis. First, we derive the theoretical conditions on the frequency separations to produce *M* symbol waveforms with negative crosscorrelation. Second, assuming equispaced frequencies, we analytically determine the optimum modulation index that maximizes the minimum distance among the symbol waveforms. Third, assuming non-equispaced frequencies, we optimize both nonorthogonal 4FSK and 8FSK signal sets. The optimized signal waveforms reduce the symbol error probability with respect to the current-best MFSK schemes existing in the literature, at the price of a bandwidth increase. For additive white Gaussian noise (AWGN) channels, an accurate expression for the symbol error probability of nonorthogonal 4FSK is also proposed.

## 1. Introduction

### 1.1. Motivation

Frequency-shift keying (FSK) is a well-established digital modulation technique, mainly used for wireless transmissions [1]. Differently from linear modulations like quadrature-amplitude modulation (QAM), the spectral efficiency of FSK reduces with increasing the number of symbols *M*; hence, FSK is mainly used for low data-rate applications. Constant envelope and low error probability are the main strengths of *M*-ary FSK (MFSK), which is suitable for low-power wireless transmissions. In the last decades, FSK and its variants have been used and proposed for a large variety of applications, including ambient backscatter communications [2,3], beamsteering [4], covert communications [5], full-duplex communications [6], integrated sensing and communication (ISAC) [7], Internet of Things (IoT) [8,9,10,11,12,13], optical networks [14], over-the-air computation [15], powerline communications (PLCs) [16,17], radar sensors [18,19,20], relay communications [21], simultaneous wireless information and power transfer (SWIPT) [22,23], space communications [24,25], underwater acoustic communications (UWACs) [26], visible light communications (VLCs) [27,28].

Typically, the *M* tones of MFSK are chosen to make the signal waveforms orthogonal, in the symbol interval [1]. In case of coherent detection, orthogonal waveforms are obtained by selecting frequencies separated by multiples of 1/(2T), where *T* is the duration of a symbol interval. However, previous studies [1] have shown that well-designed nonorthogonal waveforms can improve the error probability performance, with respect to orthogonal waveforms. Indeed, in nonorthogonal MFSK, the frequencies are not constrained to be multiple of 1/(2T) like in orthogonal MFSK; hence, there are additional degrees of freedom in the design of the signal set. These additional degrees of freedom can be exploited to increase the Euclidean distance between the *M* signal waveforms, with respect to orthogonal MFSK, thereby reducing the error probability. In addition, by exploiting the signal memory introduced by the phase continuity between consecutive waveforms, the Euclidean distance between different waveforms can be increased further: hence, a sequence detector can better distinguish the waveforms in the presence of noise, providing an additional reduction of the error probability [29,30]. However, this continuous phase FSK (CPFSK) modulation requires a sequence detector that makes decisions based on an observation window of duration NT, increasing the detection complexity to $O(M^{N})$, i.e., exponential in the window size *N*.

To reduce the complexity of the detector, while keeping a performance improvement with respect to orthogonal MFSK, one solution is to constrain the observation window size to N=1, i.e., using a symbol-by-symbol coherent detector: this coherent detector only takes advantage of the nonorthogonality of the signal waveforms, without making use of the signal memory introduced by the phase continuity [16,31]. In this case, the MFSK modulation still exploits the phase continuity for bandwidth saving purposes, but its symbol-by-symbol detector ignores the signal memory, thereby reducing the detector complexity to O(M). Therefore, a key point is how to design nonorthogonal MFSK signals that optimize the error probability performance, when the signals are detected coherently in a symbol-by-symbol way. The aim of this optimization is to quantify the maximum performance gain with respect to orthogonal MFSK. The parameters of this optimization are the *M* frequencies of the MFSK signal set, or equivalently the M−1 frequency separations of the *M* tones.

It is worth noting that coherent detection of nonorthogonal MFSK signals requires the same complexity as coherent detection of orthogonal MFSK signals. For instance, when the observation window is N=1, both orthogonal and nonorthogonal MFSK coherent detectors select the maximum among the outputs of a bank of *M* correlators that project the received signal onto the *M* signal waveforms. In both orthogonal and nonorthogonal cases, the complexity of phase recovery could be saved using noncoherent detection, at the cost of a penalty in terms of error probability. Since this paper focuses on performance optimization, noncoherent detection is not considered in this study.

### 1.2. Related Works

A first attempt of performance optimization of nonorthogonal CPFSK has been done by Schonhoff in [29], using a sequence detector that exploits the signal memory. In [29], which considers both coherent and noncoherent detection strategies, the minimization of an upper bound on the symbol error probability allows to identify the desired modulation index (and hence the desired frequency separations) for some values of alphabet size *M* and window size *N*. However, the approach of [29] has three drawbacks. First, minimizing an upper bound on the error probability may lead to a suboptimal solution, with respect to optimal solutions obtained by minimizing the exact error probability or maximizing the minimum distance among the signal waveforms. Second, the study in [29] only considers few values of *M* and does not constrain the computational complexity; therefore, the obtained results only focus on the case N>1. Third, the analysis in [29] only considers equispaced frequencies, thereby excluding potentially better solutions obtained by selecting *M* non-equispaced frequencies.

Another nonorthogonal CPFSK performance optimization for coherent detection has been done by Aulin and Sundberg in [30], which treats CPFSK as a special case of continuous phase modulation (CPM). In [30], the maximization of the minimum distance among the possible waveforms yields the desired modulation index for different values of *M* and *N*. However, the approach of [30] has two weaknesses. First, the obtained results are approximated, due to the numerical optimization, and available only for few values of *M*. Second, like in [29], the optimization in [30] does not consider the general case with *M* non-equispaced frequencies, but limits the study to the subcase with equispaced frequencies.

The study in [31], done by Sagias, Mallik and Tombras, is an important step forward in the performance optimization of MFSK. Indeed, differently from [29,30], the error probability minimization in [31] assumes *M* non-equispaced frequencies. In [31], few cases of interest have been considered, such as 4FSK and 8FSK, assuming a low-complexity coherent detector over an observation window that spans a single symbol interval. The main drawback of [31] lies in the reduced range of frequency separations spanned by the error probability minimization algorithm: the final results in [31] show that the considered range of frequency separations always produces at least one couple of positively correlated waveforms, whereas we will show that a well-designed selection of frequency separations leads to waveforms characterized by negative crosscorrelation coefficients, for all possible couples of different waveforms. Therefore, the analysis in [31] produces suboptimal results, as it will be shown in the following sections.

An analysis similar to [31] has been done in [16], assuming a specific noise model proposed for PLC systems. Specifically, a Nakagami-*m* distributed noise is assumed in [16], whereas [31] considers the popular additive white Gaussian noise (AWGN). Unfortunately, the analysis in [16] also considers a reduced subset of possible frequency separations, thereby obtaining a final solution that is again suboptimal, since there are couples of designed waveforms with positive crosscorrelation, like in [31]. Moreover, the frequency separations obtained in [16] highly depend on the noise shape parameter; therefore, the optimum transmitted waveforms depend on the receiver noise parameters, which may be unknown at the trasmitter side.

In [32], Das and Rao confirm that well-designed nonorthogonal MFSK systems outperform orthogonal MFSK schemes. However, the study in [32] focuses on rate maximization, rather than performance optimization, and therefore, there is no warranty that the MFSK scheme designed in [32] has reduced error probability with respect to the existing MFSK schemes. In addition, for rate maximization purposes, the design in [32] allows multitone MFSK waveforms obtained as the sum of tones with different frequencies; therefore, in [32], the transmitted waveforms do not have the constant-envelope advantage.

### 1.3. Contributions

In this paper, we optimize the error probability performance of MFSK in the presence of AWGN, assuming symbol-by-symbol coherent detection. In particular, we analytically design the MFSK frequency separations that maximize the minimum Euclidean distance between couples of MFSK signal waveforms, thereby minimizing the MFSK symbol error probability. Our key contributions are summarized in the following.

- First, we analytically derive the set of frequency separations that produce MFSK waveforms with negative crosscorrelation between different symbols. This analysis is carried out for the general case of non-equispaced frequencies and for the particular case of equispaced frequencies. To the best of the Author’s knowledge, this analysis is done for the first time in this paper, leading to novel mathematical conditions on the MFSK frequency separations.
- Second, for the case of MFSK with equispaced frequencies, we analytically determine the optimum modulation index that maximizes the minimum distance among the symbol waveforms. Specifically, we design the optimum MFSK for several values of the alphabet size *M*: the considered set of values is much larger than the few values considered in [29,30]. In contrast to the existing literature, our optimum MFSK schemes are derived using a clever mathematical optimization, whereas existing MFSK schemes were obtained using a computationally cumbersome numerical search. Moreover, we derive suboptimal MFSK designs for any value of *M*, and we show that the difference between optimal and suboptimal MFSK designs is negligible for M≥8. We highlight that the proposed suboptimal MFSK designs are also novel and absent in the literature. Furthermore, we show that the MFSK schemes with equispaced frequencies designed in our paper outperform the MFSK schemes with non-equispaced frequencies designed in [31]. This is an additional remarkable result of our mathematical optimization: our best MFSK scheme, even with the constraint of equispaced frequencies, outperforms the best MFSK scheme of [31] with no constraints on the frequencies; hence, our novel result contradicts the performance optimality claim previously done in [31].
- Third, differently from [29,30], we consider the more general case of non-equispaced frequencies. As specific examples, we optimize both nonorthogonal 4FSK and 8FSK signal sets. This allows a quantification of the performance gain that can be obtained with non-equispaced frequencies, with respect to equispaced frequencies only. We remark that the performance comparison between the optimal MFSK schemes is another novel contribution of our study, since the comparison between optimized equispaced frequencies and optimized non-equispaced frequencies is absent in the existing literature [29,30,31].
- Fourth, for the optimum nonorthogonal 4FSK designs, we analytically derive closed-form expressions of the symbol error probability in AWGN channels. Simulation results show that the proposed expressions are characterized by a great accuracy in both cases (either equispaced or non-equispaced frequencies). Because of the negative crosscorrelation for all couples of signal waveforms, the symbol error probability is reduced with respect to [31], where some waveform crosscorrelations are positive.

### 1.4. Paper Structure

The rest of this paper is organized as follows. Section 2 describes the transmitted signal and the received noisy signal. Section 3 derives the conditions on the frequency separations to obtain MFSK signals with negative crosscorrelation. Section 4 and Section 5 derive the optimal MFSK designs that minimize the signal crosscorrelations, assuming that the frequencies are equispaced in Section 4 and non-equispaced in Section 5. Section 6 analytically derives the symbol error probability for the optimum 4FSK schemes designed in Section 4 and Section 5. Section 7 validates the theoretical findings by comparing the performance of the proposed schemes with the existing schemes in [31]. Section 8 points out some concluding remarks.

## 2. MFSK Transmission and Reception Model

We consider a transmitter that generates equally likely MFSK waveforms expressed by

(1) $$s_{m}\left(t\right)=A\cos\left(2\pi f_{m}t+\psi \right),\;m\in \left\{1,\ldots ,M\right\},$$

in the interval 0≤t<T, where $A=\sqrt{2E_{s}/T}$ is the signal amplitude, $f_{m}$ is the frequency of the *m*th symbol waveform, ψ is the phase, *T* is the symbol period, and $E_{s}$ is the signal energy. Without loss of generality, the frequencies are arranged in increasing order, i.e., $f_{1}<f_{2}<\cdots <f_{M}$. The phase ψ, chosen to ensure phase continuity between waveforms of successive time intervals, is assumed known at the receiver. Since we consider coherent symbol-by-symbol detection, for detection purposes, we can set ψ=0 without loss of generality.

In the presence of an AWGN w(t) with power spectral density $N_{0}/2$, the received signal r(t) is expressed by

(2) $$r\left(t\right)=s_{m}\left(t\right)+w\left(t\right).$$

Since the *M* waveforms (1) are equally likely and have the same energy, the maximum-likelihood (ML) symbol-by-symbol coherent detector is a bank of *M* correlators [1], whose outputs are expressed by

(3) $$C_{n}=\int_{0}^{T}r\left(t\right)s_{n}\left(t\right)dt=\rho_{m,n}E_{s}+\int_{0}^{T}w\left(t\right)s_{n}\left(t\right)dt,\;n\in \left\{1,\ldots ,M\right\},$$

where

(4) $$\rho_{m,n}=\frac{1}{E_{s}}\int_{0}^{T}s_{m}\left(t\right)s_{n}\left(t\right)dt=\mathrm{sinc}\left(2\left(f_{n}-f_{m}\right)T\right)$$

is the crosscorrelation coefficient between the waveforms $s_{m}\left(t\right)$ and $s_{n}\left(t\right)$, while the sinc function is defined as

(5) $$\mathrm{sinc}\left(x\right)=\lim_{\varepsilon \to 0}\frac{\sin[\pi (x+\varepsilon )]}{\pi (x+\varepsilon )}.$$

The bank of correlators in (3) is then followed by a device that takes the maximum over the set $\left\{C_{n}\right\}$ to detect the transmitted symbol.

In (3), the output of the correlator with n=m, corresponding to the transmitted symbol, has a mean value $E\left\{C_{n}\right\}=\rho_{m,m}E_{s}=E_{s}$, whereas the outputs of the other correlators with n≠m, corresponding to the other M−1 symbols, have a mean value $E\left\{C_{n}\right\}=\rho_{n,m}E_{s}<E_{s}$. Therefore, the minimization of the crosscorrelation coefficients ${\left\{\rho_{m,n}\right\}}_{m\neq n}$ increases the immunity to the noise and minimizes the symbol error probability. Our aim is to select the set of *M* frequencies $\left\{f_{m}\right\}$ that minimize the crosscorrelation coefficients ${\left\{\rho_{m,n}\right\}}_{m\neq n}$ in (4). From (1), the squared Euclidean distance between two different waveforms is given by

(6) $$d_{m,n}^{2}=\int_{0}^{T}|s_{m}\left(t\right)-s_{n}{\left(t\right)|}^{2}dt=2\left(1-\rho_{m,n}\right)E_{s},$$

and hence, the minimization of the crosscorrelation coefficients ${\left\{\rho_{m,n}\right\}}_{m\neq n}$ is equivalent to the maximization of the squared Euclidean distances ${\left\{d_{m,n}^{2}\right\}}_{m\neq n}$. We define the frequency separation $\Delta_{m,n}$ as

(7) $$\Delta_{m,n}=f_{n}-f_{m}$$

and the normalized frequency separation as $h_{m,n}=\Delta_{m,n}T=\left(f_{n}-f_{m}\right)T$, so that (4) can be expressed as

(8) $$\rho_{m,n}=\mathrm{sinc}\left(2\Delta_{m,n}T\right)=\mathrm{sinc}\left(2h_{m,n}\right).$$

Consequently, the minimization of the crosscorrelation coefficients ${\left\{\rho_{m,n}\right\}}_{m\neq n}$ in (8) can be done by selecting the frequency separations $\left\{\Delta_{m,n}\right\}$ or equivalently the normalized frequency separations $\left\{h_{m,n}\right\}$.

## 3. Frequency Separations for MFSK with Negative Crosscorrelation

It is well known that orthogonal MFSK is characterized by $\rho_{m,n}=0$ for m≠n [1]. Using coherent detection, the orthogonality condition $\rho_{m,n}=0$ can be obtained by selecting equispaced frequencies with $\Delta_{m,n}=\left(n-m\right)\Delta_{1,2}=\left(n-m\right)/\left(2T\right)$. Herein, we derive the conditions on the frequency separations such that $\rho_{m,n}<0$ for all m≠n. The conditions for negative crosscorrelation coefficients can be considered as a useful preliminary step towards crosscorrelation minimization. Despite MFSK being an established digital modulation method, the general conditions for negative crosscorrelations have not been investigated in the MFSK literature, to the best of our knowledge. In addition, it is noteworthy that the 4FSK and 8FSK schemes proposed in [31], claimed to produce minimum probability of error, have some positive crosscorrelation coefficients, i.e., $\rho_{m,n}>0$ for some m≠n. Therefore, MFSK schemes with negative crosscorrelation are of interest, because they can produce improved performance with respect to both orthogonal MFSK and nonorthogonal MFSK schemes proposed in [31].

### 3.1. Sufficient Conditions for Negative Crosscorrelation

The following Theorem 1 is a sufficient condition on the normalized frequency separations $\left\{h_{m,n}\right\}$ for obtaining all-negative crosscorrelation coefficients (ANCC) ${\left\{\rho_{m,n}\right\}}_{m\neq n}$.

**Theorem 1**  (Sufficient Condition for ANCC)**.** *If*

(9) $$n-m-\frac{1}{2}<h_{m,n}<n-m$$

*for all m∈{1,…,M−1} and n∈{2,…,M} with n>m, then $\rho_{m,n}<0$ for all m≠n.*

**Proof of Theorem 1.**  Bearing in mind that sinc(x)<0 when 2k−1<|x|<2k, with *k* positive integer, from (8), the condition $\rho_{m,n}<0$ is true when $2k_{m,n}-1<2\left|h_{m,n}\right|<2k_{m,n}$, leading to

(10) $$k_{m,n}-\frac{1}{2}<h_{m,n}<k_{m,n},\;k_{m,n}\in N^{+},$$

where $h_{m,n}>0$ because of our assumption of n>m. By choosing $k_{m,n}=n-m$ in (10), we obtain the condition (9). This concludes the proof of Theorem 1. □

It is worth noting that (9) can be equivalently expressed as the intersection of M−1 conditions, listed in the following:

(11) $$\begin{array}{cc} \frac{1}{2}< & h_{m,m+1}<1,\;m\in \left\{1,\ldots ,M-1\right\}, \end{array}$$

(12) $$\begin{array}{cc} \frac{3}{2}< & h_{m,m+2}<2,\;m\in \left\{1,\ldots ,M-2\right\}, \end{array}$$

⋯

(13) $$\begin{array}{cc} M-\frac{5}{2}< & h_{m,m+M-2}<M-2,\;m\in \left\{1,2\right\}, \end{array}$$

(14) $$\begin{array}{cc} M-\frac{3}{2}< & h_{1,M}<M-1. \end{array}$$

In order to satisfy the above conditions for ANCC, the normalized frequency separations in (11)–(14) cannot be chosen independently. For instance, both $h_{m,m+1}$ and $h_{m+1,m+2}$ have to satisfy (11) and in addition, their sum $h_{m,m+2}=h_{m,m+1}+h_{m+1,m+2}$ has to satisfy (12). As an example, in 4FSK, by choosing equispaced frequencies with $h_{1,2}=h_{2,3}=0.715$, we have $\rho_{1,2}=\rho_{2,3}\approx -0.2172<0$, but $h_{1,3}=1.430$ gives $\rho_{1,3}\approx 0.0474>0$. The above counterexample proves that satisfying a single equation like (11) is not sufficient to warrant negative crosscorrelation for all couples of symbol waveforms. In any case, for any value of *M*, the intersection of (11)–(14) is not void, and hence, MFSK schemes with ANCC do exist. Indeed, the solution set always includes the following MFSK scheme, obtained by choosing equispaced frequencies with $h_{m,m+1}=\left(2M-1\right)/\left(2M\right)$, leading to $h_{m,n}=\left(n-m\right)\left(2M-1\right)/\left(2M\right)$, which satisfies all the conditions expressed by (11)–(14) or by (9). However, other solutions exist as well. In Section 5, we look for those solutions of (9) that are optimal and suboptimal from the symbol error probability viewpoint, assuming non-equispaced frequencies.

We highlight that Theorem 1 is not a necessary condition for ANCC. Sufficient conditions, different from Theorem 1, can be obtained from (10) by different selection of the integer values of $k_{m,n}$. For instance, Theorem 1 uses $k_{m,n}=n-m\leq M-1$, while a different selection could include some values with $k_{m,n}\geq M$. However, since the sinc function (5) decays toward zero for large arguments, a choice that includes $k_{m,n}\geq M$ would produce a negative crosscorrelation coefficient $\rho_{1,M}$ larger (i.e., closer to zero) than the negative crosscorrelation coefficient $\rho_{1,M}$ obtained from Theorem 1 that uses $k_{m,n}\leq M-1$. Consequently, Theorem 1 gives the sufficient condition for the most compact frequency region where to look for waveforms with the lowest-possible negative crosscorrelation coefficients, among all the sufficient conditions that can be obtained from (10).

In case of MFSK with equispaced frequencies, the frequency separations are expressed by $f_{n}-f_{m}=\left(n-m\right)\Delta$, where $\Delta =f_{m+1}-f_{m}$ is the separation between adjacent frequencies. The normalized frequency separations are given by $h_{n,m}=\left(n-m\right)h$, where h=ΔT is the modulation index. In this case, the sufficient condition (9) of Theorem 1 can be simplified, as expressed by the following Theorem 2.

**Theorem 2**  (Sufficient Condition for ANCC using Equispaced Frequencies)**.** *If*

(15) $$\frac{2M-3}{2M-2}<h<1,$$

*then $\rho_{m,n}<0$ for all m≠n.*

**Proof of Theorem 2.**  Since $h_{n,m}=\left(n-m\right)h$, the inequality in (9) becomes

(16) $$n-m-\frac{1}{2}<\left(n-m\right)h<n-m$$

or equivalently

(17) $$1-\frac{1}{2(n-m)}<h<1,$$

where n>m. The intersection of the equations in (17) for each possible value of n−m∈{1,…,M−1} produces

(18) $$\max\left\{1-\frac{1}{2(n-m)}\right\}<h<1$$

or equivalently

(19) $$1-\frac{1}{2\max\left\{n-m\right\}}<h<1.$$

The insertion of max{n−m}=M−1 into (19) yields (15) and concludes the proof of Theorem 2. □

As a consequence of Theorem 2, there exist MFSK schemes with equispaced frequencies and ANCC. Differently from designs with non-equispaced frequencies, where the choice of the multiple parameters $\left\{h_{m,n}\right\}$ must be done jointly, in Theorem 2, the single parameter *h* in (15) immediately produces a nonorthogonal MFSK scheme with ANCC. This highly simplifies the search for optimal MFSK schemes with minimum crosscorrelation, as detailed in Section 4.

### 3.2. Necessary Conditions for Negative Crosscorrelation

The following Theorem 3 is a necessary condition on the normalized frequency separations $\left\{h_{m,n}\right\}$ for ANCC ${\left\{\rho_{m,n}\right\}}_{m\neq n}$.

**Theorem 3**  (Necessary Condition for ANCC)**.** *If $\rho_{m,n}<0$ for all m≠n, where m∈{1,…,M−1} and n∈{m+1,…,M}, then*

(20) $$h_{1,M}>M-\frac{3}{2}.$$

**Proof of Theorem 3.**  We prove Theorem 3 by contradiction. If $h_{1,M}<M-3/2$, then only two cases are possible: either $M-2\leq h_{1,M}<M-3/2$, which is the first case, or $0<h_{1,M}<M-2$, which is the second case. Below, we prove that both alternative cases lead to a contradiction.

In the first case, i.e., if $M-2\leq h_{1,M}<M-3/2$, then $2M-4\leq 2h_{1,M}<2M-3$, and hence, $\rho_{1,M}=\mathrm{sinc}\left(2h_{1,M}\right)\geq 0$, because the argument $2h_{1,M}$ of the sinc function falls in an interval between an even integer and an odd integer, where the sinc function is nonnegative. Therefore, the first case contradicts the hypothesis that $\rho_{m,n}<0$ for all m≠n.

In the second case, i.e., if $0<h_{1,M}<M-2$, then $0<h_{1,2}<h_{1,3}<\ldots <h_{1,M-1}<h_{1,M}<M-2$. The inequality $\rho_{1,m}=\mathrm{sinc}\left(2h_{1,m}\right)<0$ is satisfied when $k_{m}-1/2<h_{1,m}<k_{m}$ for some $k_{m}\in \left\{1,2,\ldots ,M-2\right\}$. Hence, to satisfy $\rho_{1,m}<0$, the M−1 parameters ($h_{1,2}<h_{1,3}<\ldots <h_{1,M}$) should fall into M−2 intervals, represented by $k_{m}-1/2<h_{1,m}<k_{m}$ with $k_{m}\in \left\{1,2,\ldots ,M-2\right\}$. Being the number of intervals lower than the number of parameters, at least two parameters must fall in the same interval. Let us denote with $h_{1,\hat{m}}$ and $h_{1,\hat{m}+1}$ two consecutive parameters that fall in the same interval, with $\hat{k}=k_{\hat{m}}=k_{\hat{m}+1}$. By remembering that $h_{\hat{m},\hat{m}+1}=h_{1,\hat{m}+1}-h_{1,\hat{m}}$, we obtain $0<h_{\hat{m},\hat{m}+1}<1/2$ or equivalently $0<2h_{\hat{m},\hat{m}+1}<1$, which leads to $\rho_{\hat{m},\hat{m}+1}=\mathrm{sinc}\left(2h_{\hat{m},\hat{m}+1}\right)>0$. Therefore, the second case also contradicts the hypothesis that $\rho_{m,n}<0$ for all m≠n, thereby completing the proof of Theorem 3. □

It is worth nothing that Theorem 3 imposes a lower bound on the minimum bandwidth required by coherent MFSK with ANCC. Indeed, any reasonable definition of bandwidth *W* should include at least the *M* frequencies of the cosines in (1), thereby producing

(21) $$W\geq W_{\mathrm{LB}}=f_{M}-f_{1}=\frac{h_{1,M}}{T}>\frac{2M-3}{2T},$$

where the rightmost inequality is caused by (20). From (21), the bandwidth lower bound tends to $W_{\mathrm{LB}}\approx M/T$ for large *M*. Therefore, for large *M*, coherent nonorthogonal MFSK signals with all negative crosscorrelation coefficients must have a bandwidth approximately doubled with respect to the bandwidth $W_{\mathrm{orth}}\approx M/\left(2T\right)$ of coherent orthogonal MFSK signals with equispaced frequencies and h=1/2 [1].

In case of MFSK with equispaced frequencies, the following Theorem 4 gives a necessary condition on the modulation index *h* for negative crosscorrelation coefficients ${\left\{\rho_{m,n}\right\}}_{m\neq n}$.

**Theorem 4**  (Necessary Condition for ANCC using Equispaced Frequencies)**.** *If $\rho_{m,n}<0$ for all m≠n, then*

(22) $$h>\frac{2M-3}{2M-2}.$$

**Proof of Theorem 4.**  Since $h_{1,M}=\left(M-1\right)h$, the inequality in (20) becomes $\left(M-1\right)h>M-\frac{3}{2}$, which is equivalent to (22). Hence, the proof of Theorem 4 is completed. □

By combining Theorem 4 with Theorem 2, we can distinguish three classes of coherent nonorthogonal MFSK schemes with equispaced frequencies:

- 0<h≤(2M−3)/(2M−2), which produces at least one nonnegative crosscorrelation coefficient $\rho_{m,n}\geq 0$ with m≠n;
- (2M−3)/(2M−2)<h<1, which produces ANCC $\rho_{m,n}<0$ for all m≠n;
- h≥1, which produces a larger bandwidth than for the other two cases with h<1.

The following Section 4 focuses on the crosscorrelation minimization within the interval (2M−3)/(2M−2)<h<1, when using equispaced frequencies. In this case, the lower bound on the minimum bandwidth (21) becomes

(23) $$W\geq W_{\mathrm{LB}}=f_{M}-f_{1}=\frac{(M-1)h}{T}>\frac{2M-3}{2T}.$$

## 4. Crosscorrelation Minimization for MFSK with Equispaced Frequencies

In this section, we derive the optimal modulation index h=ΔT for nonorthogonal MFSK with equispaced frequencies $f_{n}-f_{m}=\left(n-m\right)\Delta$. We follow a minimax approach that minimizes the maximum crosscorrelation coefficient $\rho_{\max}=\max{\left\{\rho_{m,n}\right\}}_{m\neq n}$. This approach also maximizes the minimum distance between the symbol waveforms $\left\{s_{m}\left(t\right)\right\}$ in (1), with consequent benefit in terms of reduced error probability. The minimum value of the maximum crosscorrelation coefficient $\rho_{\max}$ is denoted with $\rho_{\mathrm{opt}}=\min\left\{\rho_{\max}\right\}$, where the minimization is done over all the possible modulation indexes {h}, while the modulation index that produces the minimum $\rho_{\max}=\rho_{\mathrm{opt}}$ is denoted with $h_{\mathrm{opt}}$.

Since $\rho_{m,n}=\mathrm{sinc}\left(2\left(n-m\right)h\right)$, we note that the set ${\left\{\rho_{m,n}\right\}}_{m\neq n}$ coincides with the set {sinc(2(n−m)h)} for n−m∈1,…,M−1. Then, we perform the minimization of the maximum crosscorrelation coefficient $\rho_{\max}$ in three steps: in the first step (Theorem 5), we prove that, when (2M−3)/(2M−2)<h<1, the maximum crosscorrelation coefficient is $\rho_{\max}=\rho_{1,M}$; in the second step (Theorem 6), we analytically derive the optimum modulation index $h_{\mathrm{opt}}$ that minimizes $\rho_{1,M}$ when (2M−3)/(2M−2)<h<1; in the third step (Theorem 7), we prove the uniqueness of $h_{\mathrm{opt}}$ by demonstrating that any modulation index *h* outside the interval (2M−3)/(2M−2)<h<1 produces a maximum crosscorrelation coefficient $\rho_{\max}>\rho_{\mathrm{opt}}$, which is the minimum value produced by $h_{\mathrm{opt}}$.

**Theorem 5**  (Maximum Negative Crosscorrelation Coefficient using Equispaced Frequencies)**.** *If (2M−3)/(2M−2)<h<1, then the sequence {sinc(2(n−m)h)} is negative increasing for n−m∈1,…,M−1 and consequently,*

(24) $$\rho_{\max}=\rho_{1,M}=\mathrm{sinc}\left(2\left(M-1\right)h\right).$$

**Proof of Theorem 5.**  Let us denote k=n−m and a=1−h. We have

(25) $$\mathrm{sinc}\left(2\left(n-m\right)h\right)=\mathrm{sinc}\left(2k-2ka\right)=\frac{\sin(-2\pi ka)}{2\pi k-2\pi ka}=\frac{\mathrm{sinc}\left(2ka\right)}{1-\frac{1}{a}}.$$

For fixed *h* with (2M−3)/(2M−2)<h<1, *a* is fixed with 0<a<1/(2M−2); hence, 0<2ka<k/(M−1)≤1 and 1−1/a<3−2M<0 for M≥2. Since 2ka is between zero and one, the numerator of the right-hand side of (25) is positive decreasing, while the denominator of the right-hand side (25) is a negative constant 1−1/a=−h/(1−h); hence, the sequence sinc(2ka)/(1−1/a) is negative increasing with k=n−m, with maximum value obtained for the maximum k=M−1. Consequently, $\rho_{\max}=\mathrm{sinc}\left(2\left(M-1\right)h\right)=\rho_{1,M}$, which completes the proof of Theorem 5. □

**Figure 1:**
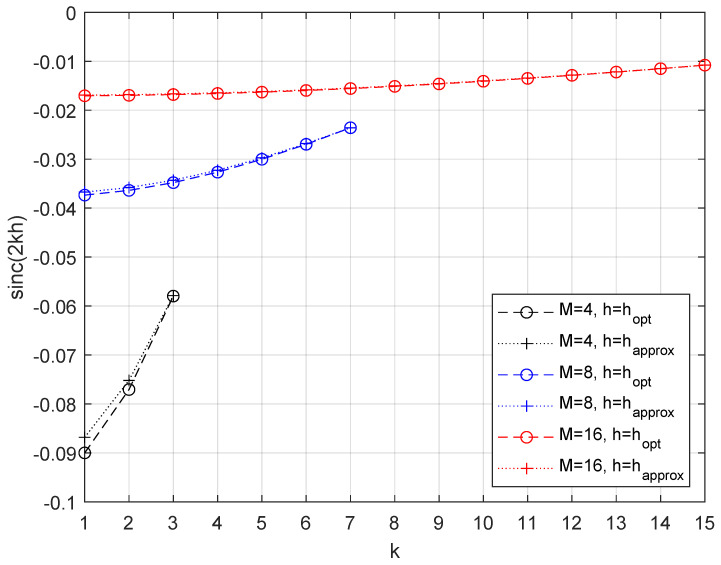
Increase of sinc(2kh) as a function of *k*, with (2M−3)/(2M−2)<h<1.

Figure 1 confirms the increasing behavior of sinc(2kh) as a function of *k*, with *k* positive integer lower than *M*, for two specific values of the modulation index *h* chosen in the interval (2M−3)/(2M−2)<h<1.

**Theorem 6**  (Optimum Modulation Index)**.** *If (2M−3)/(2M−2)<h<1, then the optimum modulation index $h_{\mathrm{opt}}$ that minimizes $\rho_{\max}$ in (24) is the unique solution of*

(26) sinc(2(M−1)h)=cos(2π(M−1)h).

**Proof of Theorem 6.**  By taking the derivative of $\rho_{\max}=\mathrm{sinc}\left(2\left(M-1\right)h\right)$ in (24) with respect to *h* and setting it to zero, we obtain

(27) $$\frac{1}{h}\left[\cos\left(2\pi \left(M-1\right)h\right)-\mathrm{sinc}\left(2\left(M-1\right)h\right)\right]=0,$$

which is equivalent to (26) and to

(28) tan(x)=x

for x=2π(M−1)h>0. Equation (28) has multiple solutions for x>0: each solution falls in an interval kπ<x<(k+1/2)π, for *k* positive integer, which corresponds to

(29) $$\frac{k}{2M-2}<h<\frac{2k+1}{4M-4}.$$

For instance, by setting k=2M−3 in (29), one solution falls in the desired interval of interest

(30) $$\frac{2M-3}{2M-2}<h<\frac{4M-5}{4M-4}<1,$$

whereas, using k=2M−2 in (29), another solution falls in the undesired interval given by

(31) $$1<h<\frac{4M-3}{4M-4}.$$

From (30) and (31), there is a unique solution of (26) in the interval (2M−3)/(2M−2)<h<1: in this interval, sinc(2(M−1)h) is negative and tends to zero at the extreme values of *h*, and therefore, the solution $h_{\mathrm{opt}}$ satisfying (26) is the unique minimum point. This completes the proof of Theorem 6. □

By combining Theorem 6 with Theorem 5, for (2M−3)/(2M−2)<h<1, the minimum value $\rho_{\mathrm{opt}}$ of the maximum crosscorrelation coefficient $\rho_{\max}$ is given by

(32) $$\rho_{\mathrm{opt}}=\mathrm{sinc}\left(2\left(M-1\right)h_{\mathrm{opt}}\right)=\cos\left(2\pi \left(M-1\right)h_{\mathrm{opt}}\right).$$

Theorem 7, at the end of this section, will prove that the optimum value $\rho_{\mathrm{opt}}$, found for $h=h_{\mathrm{opt}}$ in the interval (2M−3)/(2M−2)<h<1, is also optimum within the whole interval h>0, and that the solution $h_{\mathrm{opt}}$ given by (26) within the interval (2M−3)/(2M−2)<h<1 is the unique modulation index that achieves the minimum $\rho_{\mathrm{opt}}$ in (32).

It is worth noting that, when *M* is large, the value of the optimum modulation index $h_{\mathrm{opt}}$ is very close to

(33) $$h_{\mathrm{approx}}=\frac{4M-5}{4M-4},$$

which is the mid-value of the interval (2M−3)/(2M−2)<h<1. Because of (30), $h_{\mathrm{opt}}<h_{\mathrm{approx}}$.

Table 1 compares the optimum modulation index $h_{\mathrm{opt}}$ with its approximation $h_{\mathrm{approx}}$, and the optimum maximum crosscorrelation coefficient $\rho_{\mathrm{opt}}$ with its approximation $\rho_{\mathrm{approx}}=\mathrm{sinc}\left(2\left(M-1\right)h_{\mathrm{approx}}\right)$, for different values of *M*. The values of $h_{\mathrm{opt}}$ in Table 1, obtained by theoretical analysis and valid for detectors with single-interval observation, are similar to the few corresponding values in [30], obtained numerically and valid for detectors with multiple-interval observation. From Table 1, when M>6, the difference between the optimal values and the approximated ones is reduced, since $h_{\mathrm{approx}}-h_{\mathrm{opt}}<10^{-3}$; similarly, when M≥8, the difference between the maximum crosscorrelation values is $\rho_{\mathrm{approx}}-\rho_{\mathrm{opt}}<10^{-5}$. Therefore, the optimum modulation index $h_{\mathrm{opt}}$ calculated from (26) can be replaced by the approximated value $h_{\mathrm{approx}}$ calculated as in (33), except for small values of *M*. It is noteworthy that, when *M* increases, both $h_{\mathrm{opt}}$ and $h_{\mathrm{approx}}$ tend to 1, while both $\rho_{\mathrm{opt}}$ and $\rho_{\mathrm{approx}}$ tend to zero.

The following Theorem 7 proves the optimality of the modulation index $h_{\mathrm{opt}}$, which is unique in the whole interval h>0.

**Theorem 7**  (Uniqueness of the Optimum Modulation Index)**.** *For any $h\neq h_{\mathrm{opt}}$, the maximum crosscorrelation coefficient is*

(34) $$\rho_{\max}>\rho_{\mathrm{opt}}.$$

**Proof of Theorem 7.**  If 0<h≤(2M−3)/(2M−2), by Theorem 4, at least one crosscorrelation coefficient is $\rho_{\tilde{m},\tilde{n}}\geq 0$, hence $\rho_{\max}=\max{\left\{\rho_{m,n}\right\}}_{m\neq n}\geq \rho_{\tilde{m},\tilde{n}}\geq 0>\rho_{\mathrm{opt}}$. If (2M−3)/(2M−2)<h<1, by Theorem 6, $h_{\mathrm{opt}}$ is the unique minimum point in the interval, hence $\rho_{\max}>\rho_{\mathrm{opt}}$. If h≥1, then $h>h_{\mathrm{approx}}$, which leads to

(35) $$\rho_{\max}\geq \rho_{1,M}=\frac{\sin(2\pi (M-1)h)}{2\pi (M-1)h}\geq \frac{-1}{2\pi (M-1)h}>\frac{-1}{2\pi \left(M-1\right)h_{\mathrm{approx}}}=\rho_{\mathrm{approx}}>\rho_{\mathrm{opt}}.$$

Hence, (34) is valid for any $h\neq h_{\mathrm{opt}}$. This concludes the proof of Theorem 7. □

## 5. Crosscorrelation Minimization for MFSK with Non-Equispaced Frequencies

In this section, we search for the optimal normalized frequency separations ${\left\{h_{m,n}\right\}}_{m<n}$ for nonorthogonal MFSK with possibly non-equispaced frequencies. We follow a multi-step approach that tries to minimize all the crosscorrelation coefficients ${\left\{\rho_{m,n}\right\}}_{m<n}$. In the first step, we minimize the maximum crosscorrelation coefficient $\rho_{\max}=\max{\left\{\rho_{m,n}\right\}}_{m<n}$. The minimum value of $\rho_{\max}$ over all possible choices of ${\left\{h_{m,n}\right\}}_{m<n}$ is denoted with ${\tilde{\rho }}_{\mathrm{opt}}$. The optimum maximum crosscorrelation coefficient ${\tilde{\rho }}_{\mathrm{opt}}$ for possibly non-equispaced frequencies cannot exceed the optimum maximum crosscorrelation coefficient $\rho_{\mathrm{opt}}$ for equispaced frequencies, hence

(36) $${\tilde{\rho }}_{\mathrm{opt}}\leq \rho_{\mathrm{opt}}.$$

The second step of our approach is a further minimization to avoid ties. Indeed, in general, there exist multiple solution sets ${\left\{h_{m,n}\right\}}_{m<n}$ that produce the same ${\tilde{\rho }}_{\mathrm{opt}}$. Therefore, within these multiple solutions, the second step minimizes the second largest crosscorrelation coefficient ${\tilde{\rho }}_{\sec}=\mathrm{secmax}{\left\{\rho_{m,n}\right\}}_{m<n}$, where the operator secmax selects the second largest element in the enclosed set. In summary, the first two steps of our multi-step approach maximize both the minimum distance and the second closest distance between the symbol waveforms $\left\{s_{m}\left(t\right)\right\}$ in (1), reducing the error probability.

The first two steps may produce multiple solutions with the same minimum distance and the same second closest distance between the symbol waveforms. In this case, further optimization steps are necessary, aiming at maximizing the third closest distance, the fourth closest distance, and so on. Since MFSK signals with non-equispaced frequencies are parametrized by M−1 normalized adjacent frequency separations ${\left\{h_{m,m+1}\right\}}_{1\leq m\leq M-1}$, or equivalently by the M−1 normalized frequency separations ${\left\{h_{1,m}\right\}}_{2\leq m\leq M}$, the maximum number of required steps is M−1.

For what concerns the first step of the proposed minimization, the following Theorem 8 provides the minimum value ${\tilde{\rho }}_{\mathrm{opt}}$ of the maximum crosscorrelation coefficient $\rho_{\max}$.

**Theorem 8**  (Optimum Maximum Crosscorrelation)**.** *The minimum value ${\tilde{\rho }}_{\mathrm{opt}}$ of the maximum crosscorrelation coefficient $\rho_{\max}$ is given by*

(37) $${\tilde{\rho }}_{\mathrm{opt}}=\rho_{\mathrm{opt}},$$

*where $\rho_{\mathrm{opt}}$ in (32) is the minimum of $\rho_{\max}$ when the the M frequencies are constrained to be equispaced.*

**Proof of Theorem 8.**  First, since ${\tilde{\rho }}_{\max}=\max{\left\{\rho_{m,n}\right\}}_{m<n}$, we have

(38) $$\rho_{\max}\geq \rho_{1,M};$$

therefore, the minimization of $\rho_{\max}$ requires the minimization of $\rho_{1,M}$. By Theorem 3, a necessary condition for ANCC is $h_{1,M}>M-3/2$; therefore, the minimization of $\rho_{1,M}=\mathrm{sinc}\left(2h_{1,M}\right)$ is done over $h_{1,M}>M-3/2$. By applying the change of variable $h=h_{1,M}/\left(M-1\right)$, the minimization of $\mathrm{sinc}\left(2h_{1,M}\right)$ is equivalent to the minimization of sinc(2(M−1)h) over h>(2M−3)/(2M−2), whose solution for (2M−3)/(2M−2)<h<1 is $h=h_{\mathrm{opt}}$ given in Theorem 6. Moreover, Theorem 7 states that $h=h_{\mathrm{opt}}$ is the unique solution for h>(2M−3)/(2M−2). Therefore, the minimum value of $\rho_{1,M}$ requires $h_{1,M}=\left(M-1\right)h=\left(M-1\right)h_{\mathrm{opt}}$, which produces the minimum $\rho_{1,M}=\mathrm{sinc}\left(2h_{1,M}\right)=\mathrm{sinc}\left(2\left(M-1\right)h_{\mathrm{opt}}\right)=\cos\left(2\pi \left(M-1\right)h_{\mathrm{opt}}\right)=\rho_{\mathrm{opt}}$ by Theorem 6 and (32). By minimizing both sides of (38), we obtain

(39) $${\tilde{\rho }}_{\mathrm{opt}}\geq \rho_{\mathrm{opt}},$$

which together with (36) gives (37). This concludes the proof of Theorem 8. □

By (37), Theorem 8 states that the minimum value ${\tilde{\rho }}_{\mathrm{opt}}$ for possibly non-equispaced frequencies is equal to the minimum value $\rho_{\mathrm{opt}}$ obtained by minimization over equispaced frequencies. Therefore, the set of equispaced normalized frequencies $\left\{h_{m,n}=\left(n-m\right)h_{\mathrm{opt}}\right\}$ is a solution that produces $\rho_{\max}=\rho_{\mathrm{opt}}={\tilde{\rho }}_{\mathrm{opt}}$. By Theorem 7, this is the unique solution with equispaced frequencies. Moreover, using non-equispaced frequencies, there exist multiple solutions that produce the same minimum $\rho_{\max}=\rho_{\mathrm{opt}}={\tilde{\rho }}_{\mathrm{opt}}$ with possibly different ${\tilde{\rho }}_{\sec}$. Hence, Theorem 8 states that the optimum crosscorrelation is the same for all the multiple solutions obtained after the first step of our multi-step minimization. The following Theorem 9 gives a necessary condition on the normalized frequency separation $h_{1,M}$ for the multiple solutions that share the same $\rho_{\max}={\tilde{\rho }}_{\mathrm{opt}}$.

**Theorem 9**  (Necessary Condition for Optimum Maximum Crosscorrelation)**.** *If $\rho_{\max}={\tilde{\rho }}_{\mathrm{opt}}$, then*

(40) $$h_{1,M}=\left(M-1\right)h_{\mathrm{opt}}.$$

**Proof of Theorem 9.**  If $h_{1,M}\neq \left(M-1\right)h_{\mathrm{opt}}$, then $\rho_{1,M}=\mathrm{sinc}\left(2h_{1,M}\right)>\mathrm{sinc}\left(2\left(M-1\right)h_{\mathrm{opt}}\right)=\rho_{\mathrm{opt}}$, hence $\rho_{\max}\geq \rho_{1,M}>\rho_{\mathrm{opt}}={\tilde{\rho }}_{\mathrm{opt}}$, which contradicts the hypothesis $\rho_{\max}={\tilde{\rho }}_{\mathrm{opt}}$ of Theorem 9. This concludes the proof of Theorem 9. □

Theorem 9 states that all the solutions of the first step of the proposed multi-step minimization share the same maximum normalized frequency separation $h_{1,M}$. This reduces from M−1 to M−2 the number of degrees of freedom in the set of M(M−1)/2 normalized frequency separations ${\left\{h_{m,n}\right\}}_{m<n}$. Consequently, the maximum number of additional steps, after the first step, is M−2.

In the following subsections, we continue our proposed multi-step optimization, using an (M−2)-dimensional minimization over the normalized frequency separations ${\left\{h_{1,m}\right\}}_{2\leq m\leq M-1}$. Similarly to [31], we consider two cases of interests: M=4 and M=8. This would lead to a three-step minimization for 4FSK and to a seven-step minimization for 8FSK. However, we anticipate that the unique solution of the multi-step minimization produces non-equispaced symmetric frequency separations. Therefore, to simplify the minimization procedure, we incorporate the symmetric frequency separation constraint into the minimization problem, leading to $h_{m,n}=h_{M+1-n,M+1-m}$. Using this symmetric frequency separation constraint, the number of degrees of freedom further reduces from M−2 to M/2−1, and the number of steps of the proposed multi-step minimization reduces from M−1 to M/2. Hence, the proposed multi-step optimization is a two-step minimization for 4FSK and a four-step minimization for 8FSK. The symmetric separation constraints also reduces the number of possibly different crosscorrelation coefficients, from M(M−1)/2 to $M^{2}/4$: when M=4, the six crosscorrelation coefficients may assume up to four different values; when M=8, the 28 crosscorrelation coefficients may assume up to 16 different values.

### 5.1. Optimum 4FSK with Non-Equispaced Frequencies

Herein, we describe the proposed two-step optimization for 4FSK and derive the optimum normalized frequency separations ${\left\{h_{m,n}\right\}}_{m<n}$ that minimize both the maximum crosscorrelation coefficient and the second largest crosscorrelation coefficient. When M=4, Theorem 9 states that the optimum set of normalized frequency separations must include $h_{1,4}=3h_{\mathrm{opt}}=2.740768$, where $h_{\mathrm{opt}}$ is obtained from Table 1; otherwise, the maximum crosscorrelation coefficient $\rho_{\max}$ would be larger than the minimum value ${\tilde{\rho }}_{\mathrm{opt}}=\rho_{\mathrm{opt}}=-0.057972$ provided by Table 1. Using symmetric frequency configurations, all the normalized frequency separations ${\left\{h_{m,n}\right\}}_{m<n}$ can be expressed as a function of $h_{1,2}$ and $h_{1,4}$ only, as expressed by

(41) $$\begin{array}{cc} h_{3,4} & =h_{1,2}, \end{array}$$

(42) $$\begin{array}{cc} h_{1,3}=h_{2,4} & =h_{1,4}-h_{1,2}, \end{array}$$

(43) $$\begin{array}{cc} h_{2,3} & =h_{1,4}-2h_{1,2}. \end{array}$$

With $h_{1,4}=2.740768$ being the solution of the first optimization step, the unique variable of the second step is $h_{1,2}$.

From (11), the range of the optimization variable is $0.5<h_{1,2}<1$. This range can be reduced by imposing $\rho_{1,2}=\mathrm{sinc}\left(2h_{1,2}\right)\leq {\tilde{\rho }}_{\mathrm{opt}}=-0.057972$, which leads to $0.530977\leq h_{1,2}\leq 0.944126$. By also imposing $\rho_{2,3}=\mathrm{sinc}\left(2\left(2.740768-2h_{1,2}\right)\right)\leq {\tilde{\rho }}_{\mathrm{opt}}=-0.057972$, the range of the optimization variable can be narrowed to $0.898321\leq h_{1,2}\leq 0.944126$, which simplifies the search for the optimum $h_{1,2}$.

**Figure 2:**
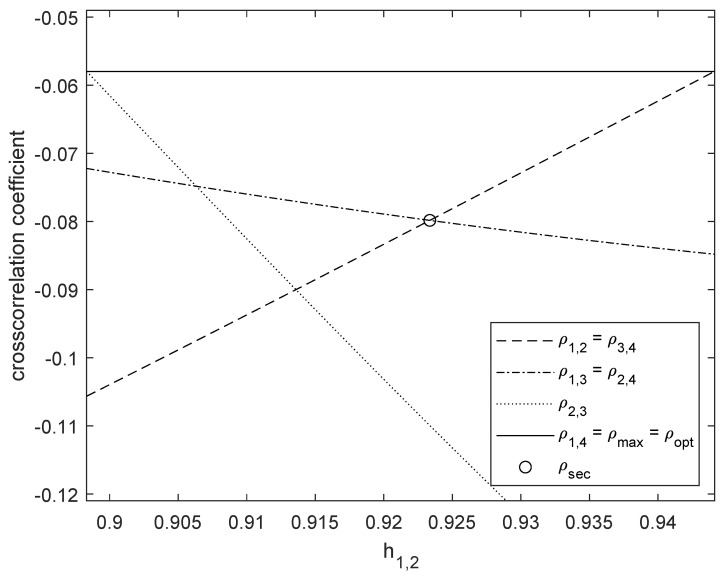
Crosscorrelation coefficients for 4FSK with $0.898321\leq h_{1,2}\leq 0.944126$ and $h_{1,4}=2.740768$.

Figure 2 shows the six crosscorrelation coefficients ${\left\{\rho_{m,n}\right\}}_{m<n}$ as a function of $h_{1,2}$, in the range where $\rho_{\max}={\tilde{\rho }}_{\mathrm{opt}}$. From Figure 2, it is clear that there is a unique value of $h_{1,2}$ that minimizes the second largest correlation coefficient. The optimum value $h_{1,2}=0.923357$ is obtained as the solution of

(44) $$\rho_{1,2}=\mathrm{sinc}\left(2h_{1,2}\right)=\mathrm{sinc}\left(2\left(h_{1,4}-h_{1,2}\right)\right)=\rho_{2,4}=\rho_{1,3},$$

where $h_{1,4}=2.740768$, in the interval $0.898321\leq h_{1,2}\leq 0.944126$. By solving (44), we obtain the optimum 4FSK scheme with non-equispaced frequencies, characterized by the following six normalized frequency separations:

(45) $$\begin{array}{cc} h_{1,2}=h_{3,4} & =0.923357, \end{array}$$

(46) $$\begin{array}{cc} h_{2,3} & =0.894054, \end{array}$$

(47) $$\begin{array}{cc} h_{1,3}=h_{2,4} & =1.817411, \end{array}$$

(48) $$\begin{array}{cc} h_{1,4} & =2.740768. \end{array}$$

The corresponding set of optimum 4FSK crosscorrelation coefficients is given by

(49) $$\begin{array}{cc} \rho_{1,4} & =-0.057972={\tilde{\rho }}_{\mathrm{opt}}, \end{array}$$

(50) $$\begin{array}{cc} \rho_{1,2}=\rho_{1,3}=\rho_{2,4}=\rho_{3,4} & =-0.079834={\tilde{\rho }}_{\sec}, \end{array}$$

(51) $$\begin{array}{cc} \rho_{2,3} & =-0.109941, \end{array}$$

leading to the correlation matrix

(52) $$R=\left(\begin{array}{cccc} 1 & -0.079834 & -0.079834 & -0.057972\\ -0.079834 & 1 & -0.109941 & -0.079834\\ -0.079834 & -0.109941 & 1 & -0.079834\\ -0.057972 & -0.079834 & -0.079834 & 1 \end{array}\right).$$

Note that, for optimum 4FSK with non-equispaced frequencies, the normalized frequency separations $h_{3,4}=h_{1,2}=0.923357$ and $h_{2,3}=0.894054$ are all different from the normalized frequency separation $h_{\mathrm{opt}}=h_{1,2}=h_{2,3}=h_{3,4}=0.913589$ derived for optimum 4FSK with equispaced frequencies. These two optimum schemes share the same $\rho_{\max}={\tilde{\rho }}_{\mathrm{opt}}=\rho_{\mathrm{opt}}=-0.057972$, but have different second largest crosscorrelation coefficient, given by ${\tilde{\rho }}_{\sec}=-0.079834$ for optimum 4FSK with non-equispaced frequencies and by $\rho_{1,3}=-0.077062$ for optimum 4FSK with equispaced frequencies. Having the same largest crosscorrelation coefficient and similar second largest crosscorrelation coefficients, it is expected that the symbol error probabilities of these two optimum schemes will be close to each other. For comparison purposes, the correlation matrix for optimum 4FSK with equispaced frequencies, where $h=h_{\mathrm{opt}}=h_{1,4}/3=0.913589$, is given by

(53) $$R=\left(\begin{array}{cccc} 1 & -0.090005 & -0.077062 & -0.057972\\ -0.090005 & 1 & -0.090005 & -0.077062\\ -0.077062 & -0.090005 & 1 & -0.090005\\ -0.057972 & -0.077062 & -0.090005 & 1 \end{array}\right).$$

In addition, both optimum 4FSK schemes derived in this paper have a maximum crosscorrelation coefficient $\rho_{\max}=-0.057972$ lower than the minimum crosscorrelation coefficient −0.0451 for the 4FSK scheme of [31]. Therefore, in the optimal 4FSK schemes derived in this paper, the distances between any symbol waveforms are all larger than the distances between any symbol waveforms for the 4FSK scheme of [31], thereby producing a reduced symbol error probability with respect to [31]. An accurate approximation for the symbol error probability of the proposed 4FSK schemes is derived in Section 6.

### 5.2. Optimum 8FSK with Non-Equispaced Frequencies

We derive the optimum normalized frequency separations ${\left\{h_{m,n}\right\}}_{m<n}$ for 8FSK. In this case, the minimization of both the maximum crosscorrelation coefficient and the second largest crosscorrelation coefficient gives multiple sets ${\left\{h_{m,n}\right\}}_{m<n}$; hence, we successively also minimize the other crosscorrelation coefficients (third largest, fourth largest, and so on): after four minimization steps, we obtain a unique solution.

The first step of the proposed four-step minimization derives from Theorem 9. When M=8, Theorem 9 states that the optimum 8FSK scheme has $h_{1,8}=7h_{\mathrm{opt}}=6.746246$, such that the maximum crosscorrelation coefficient $\rho_{\max}$ is minimized to ${\tilde{\rho }}_{\mathrm{opt}}=\rho_{\mathrm{opt}}=-0.023585$ provided by Table 1. Using symmetrical frequency configurations, all the normalized frequency separations ${\left\{h_{m,n}\right\}}_{m<n}$ can be expressed as a function of the four parameters $h_{1,2}$, $h_{2,3}$, $h_{3,4}$, and $h_{1,8}$, because all the other normalized frequency separations can be expressed as a function of the above four parameters:

(54) $$\begin{array}{cc} h_{4,5} & =h_{1,8}-2h_{1,2}-2h_{2,3}-2h_{3,4}, \end{array}$$

(55) $$\begin{array}{cc} h_{5,6} & =h_{3,4}, \end{array}$$

(56) $$\begin{array}{cc} h_{6,7} & =h_{2,3}, \end{array}$$

(57) $$\begin{array}{cc} h_{7,8} & =h_{1,2}, \end{array}$$

(58) $$\begin{array}{cc} h_{1,3}=h_{6,8} & =h_{1,2}+h_{2,3}, \end{array}$$

(59) $$\begin{array}{cc} h_{2,4}=h_{5,7} & =h_{2,3}+h_{3,4}, \end{array}$$

(60) $$\begin{array}{cc} h_{3,5}=h_{4,6} & =h_{1,8}-2h_{1,2}-2h_{2,3}-h_{3,4}, \end{array}$$

(61) $$\begin{array}{cc} h_{1,4}=h_{5,8} & =h_{1,2}+h_{2,3}+h_{3,4}, \end{array}$$

(62) $$\begin{array}{cc} h_{2,5}=h_{4,7} & =h_{1,8}-2h_{1,2}-h_{2,3}-h_{3,4}, \end{array}$$

(63) $$\begin{array}{cc} h_{3,6} & =h_{1,8}-2h_{1,2}-2h_{2,3}, \end{array}$$

(64) $$\begin{array}{cc} h_{1,5}=h_{4,8} & =h_{1,8}-h_{1,2}-h_{2,3}-h_{3,4}, \end{array}$$

(65) $$\begin{array}{cc} h_{2,6}=h_{3,7} & =h_{1,8}-2h_{1,2}-h_{2,3}, \end{array}$$

(66) $$\begin{array}{cc} h_{1,6}=h_{3,8} & =h_{1,8}-h_{1,2}-h_{2,3}, \end{array}$$

(67) $$\begin{array}{cc} h_{2,7} & =h_{1,8}-2h_{1,2}, \end{array}$$

(68) $$\begin{array}{cc} h_{1,7}=h_{2,8} & =h_{1,8}-h_{1,2}. \end{array}$$

Being $h_{1,8}=6.746246$, the three variables of the last three steps of the 8FSK optimization are $h_{1,2}$, $h_{2,3}$, $h_{3,4}$. From (11), the range of the three optimization variables is $0.5<h_{m,m+1}<1$. This range can be reduced by imposing $\rho_{m,m+1}\leq {\tilde{\rho }}_{\mathrm{opt}}=\mathrm{sinc}\left(2h_{1,8}\right)=-0.023585$.

The second step of the proposed four-step minimization for 8FSK is similar to the second step already described for 4FSK. Like in (44), the second largest crosscorrelation coefficient is minimized when

(69) $$\rho_{1,2}=\mathrm{sinc}\left(2h_{1,2}\right)=\mathrm{sinc}\left(2\left(h_{1,8}-h_{1,2}\right)\right)=\rho_{2,8}=\rho_{1,7},$$

where $h_{1,8}=6.746246$, thereby obtaining the optimum 8FSK normalized frequency separations

(70) $$\begin{array}{cc} h_{1,2}=h_{7,8} & =0.973320, \end{array}$$

(71) $$\begin{array}{cc} h_{2,7} & =4.799606, \end{array}$$

(72) $$\begin{array}{cc} h_{1,7}=h_{2,8} & =5.772926, \end{array}$$

(73) $$\begin{array}{cc} h_{1,8} & =6.746246, \end{array}$$

and the optimum 8FSK crosscorrelation coefficients

(74) $$\begin{array}{cc} \rho_{1,8} & =-0.023585={\tilde{\rho }}_{\mathrm{opt}}, \end{array}$$

(75) $$\begin{array}{cc} \rho_{1,2}=\rho_{1,7}=\rho_{2,8}=\rho_{7,8} & =-0.027283={\tilde{\rho }}_{\sec}, \end{array}$$

(76) $$\begin{array}{cc} \rho_{2,7} & =-0.031562. \end{array}$$

The other normalized frequency separations are obtained by further minimization of the next largest crosscorrelation coefficients, excluding the above crosscorrelation coefficients already optimized. Specifically, the third minimization step for optimum 8FSK design is solved as follows. Using $h_{1,8}=6.746246$ and $h_{1,2}=0.973320$, similarly to (69), the third largest crosscorrelation coefficient is minimized when

(77) $$\rho_{1,3}=\mathrm{sinc}\left(2h_{1,3}\right)=\mathrm{sinc}\left(2\left(h_{1,8}-h_{1,3}\right)\right)=\rho_{3,8}=\rho_{1,6},$$

thereby obtaining other optimum 8FSK normalized frequency separations

(78) $$\begin{array}{cc} h_{2,3}=h_{6,7} & =0.965168, \end{array}$$

(79) $$\begin{array}{cc} h_{1,3}=h_{6,8} & =1.938488, \end{array}$$

(80) $$\begin{array}{cc} h_{3,6} & =2.869270, \end{array}$$

(81) $$\begin{array}{cc} h_{2,6}=h_{3,7} & =3.834438 \end{array}$$

(82) $$\begin{array}{cc} h_{1,6}=h_{3,8} & =4.807758, \end{array}$$

and the corresponding crosscorrelation coefficients

(83) $$\begin{array}{cc} \rho_{1,3}=\rho_{1,6}=\rho_{3,8}=\rho_{6,8} & =-0.030948, \end{array}$$

(84) $$\begin{array}{cc} \rho_{2,3}=\rho_{2,6}=\rho_{3,7}=\rho_{6,7} & =-0.035801, \end{array}$$

(85) $$\begin{array}{cc} \rho_{3,6} & =-0.040609. \end{array}$$

Finally, the fourth minimization step is performed: using $h_{1,8}=6.746246$, $h_{1,2}=0.973320$, and $h_{2,3}=0.965168$, we minimize again the largest crosscorrelation coefficient, among the coefficients still undetermined. The solution of this final minimization is given by

(86) $$\rho_{1,4}=\mathrm{sinc}\left(2h_{1,4}\right)=\mathrm{sinc}\left(2\left(h_{1,8}-h_{1,4}\right)\right)=\rho_{4,8}=\rho_{1,5},$$

which gives the remaining optimum 8FSK normalized frequency separations

(87) $$\begin{array}{cc} h_{3,4}=h_{5,6} & =0.957525, \end{array}$$

(88) $$\begin{array}{cc} h_{4,5} & =0.954220, \end{array}$$

(89) $$\begin{array}{cc} h_{2,4}=h_{5,7} & =1.922693, \end{array}$$

(90) $$\begin{array}{cc} h_{3,5}=h_{4,6} & =1.911745, \end{array}$$

(91) $$\begin{array}{cc} h_{1,4}=h_{5,8} & =2.896013, \end{array}$$

(92) $$\begin{array}{cc} h_{2,5}=h_{4,7} & =2.876913, \end{array}$$

(93) $$\begin{array}{cc} h_{1,5}=h_{4,8} & =3.850233, \end{array}$$

and the corresponding crosscorrelation coefficients

(94) $$\begin{array}{cc} \rho_{1,4}=\rho_{1,5}=\rho_{4,8}=\rho_{5,8} & =-0.033406, \end{array}$$

(95) $$\begin{array}{cc} \rho_{2,4}=\rho_{2,5}=\rho_{4,7}=\rho_{5,7} & =-0.038645, \end{array}$$

(96) $$\begin{array}{cc} \rho_{3,4}=\rho_{3,5}=\rho_{4,6}=\rho_{5,6} & =-0.043834, \end{array}$$

(97) $$\begin{array}{cc} \rho_{4,5} & =-0.047318. \end{array}$$

Note that this fourth step actually minimizes the fifth largest crosscorrelation coefficient $\rho_{1,4}=-0.033406$, since the fourth largest crosscorrelation coefficient $\rho_{2,7}=-0.031562$ was already determined during the second step. It is also worth noting that the optimum 8FSK frequency separations are larger at the edges of the band, with $h_{1,2}=h_{7,8}=0.973320$, while the frequency separation is lower in the center band, with $h_{4,5}=0.954220$. Summarizing, the correlation matrix for the optimum 8FSK with non-equispaced frequencies is given by

(98) $$R\approx \left(\begin{array}{cccccccc} 1 & -0.0273 & -0.0309 & -0.0334 & -0.0334 & -0.0309 & -0.0273 & -0.0236\\ -0.0273 & 1 & -0.0358 & -0.0386 & -0.0386 & -0.0358 & -0.0316 & -0.0273\\ -0.0309 & -0.0358 & 1 & -0.0438 & -0.0438 & -0.0406 & -0.0358 & -0.0309\\ -0.0334 & -0.0386 & -0.0438 & 1 & -0.0473 & -0.0438 & -0.0386 & -0.0334\\ -0.0334 & -0.0386 & -0.0438 & -0.0473 & 1 & -0.0438 & -0.0386 & -0.0334\\ -0.0309 & -0.0358 & -0.0406 & -0.0438 & -0.0438 & 1 & -0.0358 & -0.0309\\ -0.0273 & -0.0316 & -0.0358 & -0.0386 & -0.0386 & -0.0358 & 1 & -0.0273\\ -0.0236 & -0.0273 & -0.0309 & -0.0334 & -0.0334 & -0.0309 & -0.0273 & 1 \end{array}\right).$$

For comparison purposes, the correlation matrix for optimum 8FSK with equispaced frequencies, where $h=h_{\mathrm{opt}}=h_{1,8}/7=0.963749$, is given by

(99) $$R\approx \left(\begin{array}{cccccccc} 1 & -0.0373 & -0.0363 & -0.0348 & -0.0326 & -0.0300 & -0.0270 & -0.0236\\ -0.0373 & 1 & -0.0373 & -0.0363 & -0.0348 & -0.0326 & -0.0300 & -0.0270\\ -0.0363 & -0.0373 & 1 & -0.0373 & -0.0363 & -0.0348 & -0.0326 & -0.0300\\ -0.0348 & -0.0363 & -0.0373 & 1 & -0.0373 & -0.0363 & -0.0348 & -0.0326\\ -0.0326 & -0.0348 & -0.0363 & -0.0373 & 1 & -0.0373 & -0.0363 & -0.0348\\ -0.0300 & -0.0326 & -0.0348 & -0.0363 & -0.0373 & 1 & -0.0373 & -0.0363\\ -0.0270 & -0.0300 & -0.0326 & -0.0348 & -0.0363 & -0.0373 & 1 & -0.0373\\ -0.0236 & -0.0270 & -0.0300 & -0.0326 & -0.0348 & -0.0363 & -0.0373 & 1 \end{array}\right).$$

Both (98) and (99) confirm that the correlation matrix (for both optimum 8FSK schemes) contains only negative elements outside the main diagonal, whereas in [31], some elements outside the main diagonal are positive. Therefore, it is expected that both the designed 8FSK schemes outperform the existing 8FSK scheme of [31], in terms of symbol error probability.

### 5.3. Optimum MFSK with Non-Equispaced Frequencies

We now generalize our multi-step optimization to the cases with M>8. Let us assume *M* a power of two. By using the same procedure for 4FSK and 8FSK described in Section 5.1 and Section 5.2, all the normalized frequency separations can be obtained using a sequence of M/2 successive minimization steps. In the first step, the maximum separation $h_{1,M}$ is determined as $h_{1,M}=\left(M-1\right)h_{\mathrm{opt}}$, with $h_{\mathrm{opt}}$ given by Table 1. If M>256, $h_{1,M}$ may be approximated by $h_{1,M}\approx \left(M-1\right)h_{\mathrm{approx}}=M-1.25$, using $h_{\mathrm{approx}}$ calculated as in (33). Then, similarly to (69), (77) and (86), the normalized frequency separations are obtained by solving sequentially, for i=2,…,M/2, the equation

(100) $$\rho_{1,i}=\mathrm{sinc}\left(2h_{1,i}\right)=\mathrm{sinc}\left(2\left(h_{1,M}-h_{1,i}\right)\right)=\rho_{i,M},$$

where *i* also denotes the index of the step. This multi-step optimization allows to sequentially determine the normalized frequency separations $\left\{h_{1,i}\right\}$ for i=2,…,M/2. All the other normalized frequency separations can be obtained from the ones already derived, including $h_{1,M}$ derived in the first step, and exploiting the symmetry constraint.

## 6. Symbol Error Probability for Optimum 4FSK

In this section, we present an accurate approximation of the symbol error probability for the optimum nonorthogonal 4FSK schemes derived in Section 4 and Section 5.1. We assume M=4 equiprobable waveforms, the noise model in (2), and a maximum-likelihood coherent receiver over a single observation interval of duration *T*.

To derive the four constellation vectors, we apply the Gram–Schmidt orthonormalization procedure [1] to the four nonorthogonal signal waveforms in (1). By defining

(101) $$\begin{array}{cc} R_{2} & =\sqrt{1-\rho_{1,2}^{2}}\;, \end{array}$$

(102) $$\begin{array}{cc} u_{2,3} & =\frac{\rho_{2,3}-\rho_{1,2}\rho_{1,3}}{R_{2}}\;, \end{array}$$

(103) $$\begin{array}{cc} R_{3} & =\sqrt{1-\rho_{1,3}^{2}-u_{2,3}^{2}}\;, \end{array}$$

(104) $$\begin{array}{cc} u_{2,4} & =\frac{\rho_{2,4}-\rho_{1,2}\rho_{1,4}}{R_{2}}\;, \end{array}$$

(105) $$\begin{array}{cc} u_{3,4} & =\frac{\rho_{3,4}-\rho_{1,3}\rho_{1,4}-u_{2,3}u_{2,4}}{R_{3}}\;, \end{array}$$

(106) $$\begin{array}{cc} R_{4} & =\sqrt{1-\rho_{1,4}^{2}-u_{2,4}^{2}-u_{3,4}^{2}}\;, \end{array}$$

we obtain the orthonormal expansion of the *m*th signal waveform as

(107) $$s_{m}\left(t\right)=\sum_{n=1}^{4}s_{m,n}\phi_{n}\left(t\right),$$

where $\phi_{n}\left(t\right)$ is the *n*th orthonormal function of the nonorthogonal 4FSK signal space, expressed by

(108) $$\begin{array}{cc} \phi_{1}\left(t\right) & =\frac{1}{\sqrt{E_{s}}}s_{1}\left(t\right)\;, \end{array}$$

(109) $$\begin{array}{cc} \phi_{2}\left(t\right) & =\frac{1}{\sqrt{E_{s}}R_{2}}s_{2}\left(t\right)-\frac{\rho_{1,2}}{R_{2}}\phi_{1}\left(t\right) \end{array}$$

(110) $$\begin{array}{cc} \; & =-\frac{\rho_{1,2}}{\sqrt{E_{s}}R_{2}}s_{1}\left(t\right)+\frac{1}{\sqrt{E_{s}}R_{2}}s_{2}\left(t\right)\;, \end{array}$$

(111) $$\begin{array}{cc} \phi_{3}\left(t\right) & =\frac{1}{\sqrt{E_{s}}R_{3}}s_{3}\left(t\right)-\frac{\rho_{1,3}}{R_{3}}\phi_{1}\left(t\right)-\frac{u_{2,3}}{R_{3}}\phi_{2}\left(t\right) \end{array}$$

(112) $$\begin{array}{cc} \; & =-\frac{\rho_{1,3}-R_{2}^{-1}\rho_{1,2}u_{2,3}}{\sqrt{E_{s}}R_{3}}s_{1}\left(t\right)-\frac{R_{2}^{-1}u_{2,3}}{\sqrt{E_{s}}R_{3}}s_{2}\left(t\right)+\frac{1}{\sqrt{E_{s}}R_{3}}s_{3}\left(t\right)\;, \end{array}$$

(113) $$\begin{array}{cc} \phi_{4}\left(t\right) & =\frac{1}{\sqrt{E_{s}}R_{4}}s_{4}\left(t\right)-\frac{\rho_{1,4}}{R_{4}}\phi_{1}\left(t\right)-\frac{u_{2,4}}{R_{4}}\phi_{2}\left(t\right)-\frac{u_{3,4}}{R_{4}}\phi_{3}\left(t\right) \end{array}$$

(114) $$\begin{array}{cc} \; & =-\frac{\pi_{4,1}}{\sqrt{E_{s}}R_{4}}s_{1}\left(t\right)-\frac{\pi_{4,2}}{\sqrt{E_{s}}R_{4}}s_{2}\left(t\right)-\frac{R_{3}^{-1}u_{3,4}}{\sqrt{E_{s}}R_{4}}s_{3}\left(t\right)+\frac{1}{\sqrt{E_{s}}R_{4}}s_{4}\left(t\right)\;, \end{array}$$

with

(115) $$\begin{array}{cc} \pi_{4,1} & =\rho_{1,4}-R_{2}^{-1}\rho_{1,2}u_{2,4}-R_{3}^{-1}\rho_{1,3}u_{3,4}+R_{2}^{-1}R_{3}^{-1}\rho_{1,2}u_{2,3}u_{3,4}\;, \end{array}$$

(116) $$\begin{array}{cc} \pi_{4,2} & =R_{2}^{-1}u_{2,4}-R_{2}^{-1}R_{3}^{-1}u_{2,3}u_{3,4}\;, \end{array}$$

while $s_{m,n}$ in (107) is the *n*th coefficient of the *m*th constellation vector $s_{m}$ expressed by

(117) $$\begin{array}{cc} s_{1} & =\sqrt{E_{s}}{\left(\begin{array}{cccc} 1 & 0 & 0 & 0 \end{array}\right)}^{T}\;, \end{array}$$

(118) $$\begin{array}{cc} s_{2} & =\sqrt{E_{s}}{\left(\begin{array}{cccc} \rho_{1,2} & R_{2} & 0 & 0 \end{array}\right)}^{T}\;, \end{array}$$

(119) $$\begin{array}{cc} s_{3} & =\sqrt{E_{s}}{\left(\begin{array}{cccc} \rho_{1,3} & u_{2,3} & R_{3} & 0 \end{array}\right)}^{T}\;, \end{array}$$

(120) $$\begin{array}{cc} s_{4} & =\sqrt{E_{s}}{\left(\begin{array}{cccc} \rho_{1,4} & u_{2,4} & u_{3,4} & R_{4} \end{array}\right)}^{T}\;. \end{array}$$

Using the basis expansion (107)–(116) and the constellation vectors (117)–(120), the system Equation (2) leads to the received vector

(121) $$r=s_{m}+w\;,$$

where $s_{m}$ is the transmitted vector and w is a jointly Gaussian real-valued vector with zero mean and covariance $\frac{N_{0}}{2}I_{4}$.

For equienergy signals like MFSK signals, the maximum-likelihood coherent receiver calculates the *M* correlations between the received vector r and the *M* symbol vectors ${\left\{s_{i}\right\}}_{1\leq i\leq M}$ and then selects the symbol with maximum value among the *M* correlator outputs ${\left\{s_{i}^{T}r\right\}}_{1\leq i\leq M}$. Hence, the decision region of $s_{1}$ is given by $R_{1}=\left\{r:\left(s_{1}^{T}r>s_{2}^{T}r\right)\wedge \left(s_{1}^{T}r>s_{3}^{T}r\right)\wedge \left(s_{1}^{T}r>s_{4}^{T}r\right)\right\}$, while the decision regions $R_{2}$, $R_{3}$, and $R_{4}$ are similarly defined. The probability of symbol error $P_{e}$ is obtained as

(122) $$P_{e}=\frac{1}{4}\sum_{n=1}^{4}\mathrm{Pr}\{r\notin R_{n}|s_{m}=s_{n}\}\;,$$

where

(123) $$\begin{array}{cc} \mathrm{Pr}\{r\notin R_{1}|s_{m} & =s_{1}\}\;=\mathrm{Pr}\left\{E_{1,2}\cup E_{1,3}\cup E_{1,4}\right\}\;, \end{array}$$

(124) $$\begin{array}{cc} \mathrm{Pr}\{r\notin R_{2}|s_{m} & =s_{2}\}\;=\mathrm{Pr}\left\{E_{2,1}\cup E_{2,3}\cup E_{2,4}\right\}\;, \end{array}$$

(125) $$\begin{array}{cc} \mathrm{Pr}\{r\notin R_{3}|s_{m} & =s_{3}\}\;=\mathrm{Pr}\left\{E_{3,1}\cup E_{3,2}\cup E_{3,4}\right\}\;, \end{array}$$

(126) $$\begin{array}{cc} \mathrm{Pr}\{r\notin R_{4}|s_{m} & =s_{4}\}\;=\mathrm{Pr}\left\{E_{4,1}\cup E_{4,2}\cup E_{4,3}\right\}\;, \end{array}$$

where the event $E_{n,i}$ is the pairwise error event when the transmitted symbol is $s_{n}$ but the received symbol is $s_{i}$. Using a Bonferroni identity, the conditional probabilities (123)–(126) can be expressed as

(127) $$\begin{array}{cc} \mathrm{Pr}\{E_{1,2}\cup E_{1,3}\cup E_{1,4}\} & =\mathrm{Pr}\left\{E_{1,2}\right\}+\mathrm{Pr}\left\{E_{1,3}\right\}+\mathrm{Pr}\left\{E_{1,4}\right\}+\mathrm{Pr}\left\{E_{1,2}\cap E_{1,3}\cap E_{1,4}\right\}\\ & -\mathrm{Pr}\left\{E_{1,2}\cap E_{1,3}\right\}-\mathrm{Pr}\left\{E_{1,2}\cap E_{1,4}\right\}-\mathrm{Pr}\left\{E_{1,3}\cap E_{1,4}\right\}\;, \end{array}$$

(128) $$\begin{array}{cc} \mathrm{Pr}\{E_{2,1}\cup E_{2,3}\cup E_{2,4}\} & =\mathrm{Pr}\left\{E_{2,1}\right\}+\mathrm{Pr}\left\{E_{2,3}\right\}+\mathrm{Pr}\left\{E_{2,4}\right\}+\mathrm{Pr}\left\{E_{2,1}\cap E_{2,3}\cap E_{2,4}\right\}\\ & -\mathrm{Pr}\left\{E_{2,1}\cap E_{2,3}\right\}-\mathrm{Pr}\left\{E_{2,1}\cap E_{2,4}\right\}-\mathrm{Pr}\left\{E_{2,3}\cap E_{2,4}\right\}\;, \end{array}$$

(129) $$\begin{array}{cc} \mathrm{Pr}\{E_{3,1}\cup E_{3,2}\cup E_{3,4}\} & =\mathrm{Pr}\left\{E_{3,1}\right\}+\mathrm{Pr}\left\{E_{3,2}\right\}+\mathrm{Pr}\left\{E_{3,4}\right\}+\mathrm{Pr}\left\{E_{3,1}\cap E_{3,2}\cap E_{3,4}\right\}\\ & -\mathrm{Pr}\left\{E_{3,1}\cap E_{3,2}\right\}-\mathrm{Pr}\left\{E_{3,1}\cap E_{3,4}\right\}-\mathrm{Pr}\left\{E_{3,2}\cap E_{3,4}\right\}\;, \end{array}$$

(130) $$\begin{array}{cc} \mathrm{Pr}\{E_{4,1}\cup E_{4,2}\cup E_{4,3}\} & =\mathrm{Pr}\left\{E_{4,1}\right\}+\mathrm{Pr}\left\{E_{4,2}\right\}+\mathrm{Pr}\left\{E_{4,3}\right\}+\mathrm{Pr}\left\{E_{4,1}\cap E_{4,2}\cap E_{4,3}\right\}\\ & -\mathrm{Pr}\left\{E_{4,1}\cap E_{4,2}\right\}-\mathrm{Pr}\left\{E_{4,1}\cap E_{4,3}\right\}-\mathrm{Pr}\left\{E_{4,2}\cap E_{4,3}\right\}\;. \end{array}$$

By inserting (123)–(130) into (122), the symbol error probability can be expressed as

(131) $$P_{e}=U-\frac{K}{4}\;,$$

where

(132) $$U=\frac{1}{4}\sum_{n=1}^{4}\sum_{i\neq n}\mathrm{Pr}\{E_{n,i}\}$$

is the upper bound obtained using the union bound, while

(133) $$\begin{array}{cc} K & =\mathrm{Pr}\left\{E_{1,2}\cap E_{1,3}\right\}+\mathrm{Pr}\left\{E_{1,2}\cap E_{1,4}\right\}+\mathrm{Pr}\left\{E_{1,3}\cap E_{1,4}\right\}\\ \; & +\mathrm{Pr}\left\{E_{2,1}\cap E_{2,3}\right\}+\mathrm{Pr}\left\{E_{2,1}\cap E_{2,4}\right\}+\mathrm{Pr}\left\{E_{2,3}\cap E_{2,4}\right\}\\ \; & +\mathrm{Pr}\left\{E_{3,1}\cap E_{3,2}\right\}+\mathrm{Pr}\left\{E_{3,1}\cap E_{3,4}\right\}+\mathrm{Pr}\left\{E_{3,2}\cap E_{3,4}\right\}\\ \; & +\mathrm{Pr}\left\{E_{4,1}\cap E_{4,2}\right\}+\mathrm{Pr}\left\{E_{4,1}\cap E_{4,3}\right\}+\mathrm{Pr}\left\{E_{4,2}\cap E_{4,3}\right\}\\ \; & -\mathrm{Pr}\left\{E_{1,2}\cap E_{1,3}\cap E_{1,4}\right\}-\mathrm{Pr}\left\{E_{2,1}\cap E_{2,3}\cap E_{2,4}\right\}\\ \; & -\mathrm{Pr}\left\{E_{3,1}\cap E_{3,2}\cap E_{3,4}\right\}-\mathrm{Pr}\left\{E_{4,1}\cap E_{4,2}\cap E_{4,3}\right\} \end{array}$$

is the quadruple correction term necessary for subtracting the errors that have been counted twice in the union bound (132). The pairwise error event $E_{n,i}$ in (132) is equivalent to the event

(134) $$s_{n}^{T}\left(s_{n}+w\right)<s_{i}^{T}\left(s_{n}+w\right),$$

where the *n*th correlator output is less than the *i*th correlator output, conditioned on the transmitted symbol $s_{m}=s_{n}$. The same event $E_{n,i}$ corresponds to

(135) $$E_{s}\left(1-\rho_{n,i}\right)<{\left(s_{i}-s_{n}\right)}^{T}w,$$

whose probability is given by

(136) $$\mathrm{Pr}\left\{E_{n,i}\right\}=Q\left(\sqrt{\left(1-\rho_{n,i}\right)\frac{E_{s}}{N_{0}}}\right)\;,$$

where Q(x) is the Gaussian Q-function, and hence, the union bound term *U* in (132) is given by

(137) $$U=\frac{1}{4}\sum_{n=1}^{4}\sum_{i\neq n}Q\left(\sqrt{\left(1-\rho_{n,i}\right)\frac{E_{s}}{N_{0}}}\right)=\frac{1}{2}\sum_{n=1}^{3}\sum_{i=n+1}^{4}Q\left(\sqrt{\left(1-\rho_{n,i}\right)\frac{E_{s}}{N_{0}}}\right)\;.$$

For what concerns the quadruple correction term *K* in (133), we can exploit the accurate approximation of [33], valid for quaternary equicorrelated equienergy signals. The optimum nonorthogonal 4FSK schemes designed in Section 4 and Section 5.1 are characterized by signals with different crosscorrelation coefficients. However, for both correlation matrices (52) and (53), the differences between the crosscorrelation coefficients in the same matrix are limited, since the maximum value is −0.057972 and the minimum value is either −0.109941 or −0.090005. Therefore, for computing an approximation of the correction term *K*, the small differences between the crosscorrelation coefficients can be neglected. By defining the average crosscorrelation coefficient $\rho_{\mathrm{avg}}$ as

(138) $$\rho_{\mathrm{avg}}=\frac{1}{6}\left(\rho_{1,2}+\rho_{1,3}+\rho_{1,4}+\rho_{2,3}+\rho_{2,4}+\rho_{3,4}\right)\;,$$

using the result of [33], the quadruple correction term can be approximated as

(139) $$K\approx 52Q\left(\sqrt{\left(1-\rho_{\mathrm{avg}}\right)\frac{E_{s}}{N_{0}}}\right)Q\left(\sqrt{\left(1-\rho_{\mathrm{avg}}\right)\frac{E_{s}}{3N_{0}}}\right)-40Q^{2}\left(\sqrt{\left(1-\rho_{\mathrm{avg}}\right)\frac{2E_{s}}{3N_{0}}}\right)\;.$$

Summarizing, by inserting (137) and (139) in (131), the symbol error probability of the designed optimum nonorthogonal 4FSK with non-equispaced frequencies can be well approximated by

(140) $$\begin{array}{cc} P_{e} & \approx \frac{1}{2}Q\left(\sqrt{\left(1-\rho_{1,2}\right)\frac{E_{s}}{N_{0}}}\right)+\frac{1}{2}Q\left(\sqrt{\left(1-\rho_{1,3}\right)\frac{E_{s}}{N_{0}}}\right)+\frac{1}{2}Q\left(\sqrt{\left(1-\rho_{1,4}\right)\frac{E_{s}}{N_{0}}}\right)\\ \; & +\frac{1}{2}Q\left(\sqrt{\left(1-\rho_{2,3}\right)\frac{E_{s}}{N_{0}}}\right)+\frac{1}{2}Q\left(\sqrt{\left(1-\rho_{2,4}\right)\frac{E_{s}}{N_{0}}}\right)+\frac{1}{2}Q\left(\sqrt{\left(1-\rho_{3,4}\right)\frac{E_{s}}{N_{0}}}\right)\\ \; & -13Q\left(\sqrt{\left(1-\rho_{\mathrm{avg}}\right)\frac{E_{s}}{N_{0}}}\right)Q\left(\sqrt{\left(1-\rho_{\mathrm{avg}}\right)\frac{E_{s}}{3N_{0}}}\right)+10Q^{2}\left(\sqrt{\left(1-\rho_{\mathrm{avg}}\right)\frac{2E_{s}}{3N_{0}}}\right)\;, \end{array}$$

where $\rho_{\mathrm{avg}}$ is given in (138). The optimum nonorthogonal 4FSK scheme with non-equispaced frequencies derived in Section 5.1 has $\rho_{\mathrm{avg}}=-0.081208$; hence, the symbol error probability (140) becomes

(141) $$\begin{array}{cc} P_{e} & \approx \frac{1}{2}Q\left(\sqrt{1.057972\frac{E_{s}}{N_{0}}}\right)+2Q\left(\sqrt{1.079834\frac{E_{s}}{N_{0}}}\right)+\frac{1}{2}Q\left(\sqrt{1.109941\frac{E_{s}}{N_{0}}}\right)\\ \; & -13Q\left(\sqrt{1.081208\frac{E_{s}}{N_{0}}}\right)Q\left(\sqrt{0.360403\frac{E_{s}}{N_{0}}}\right)+10Q^{2}\left(\sqrt{0.720805\frac{E_{s}}{N_{0}}}\right)\;. \end{array}$$

On the other hand, the optimum nonorthogonal 4FSK scheme with equispaced frequences derived in Section 4 has $\rho_{\mathrm{avg}}=-0.080352$; hence, the symbol error probability is given by

(142) $$\begin{array}{cc} P_{e} & \approx \frac{1}{2}Q\left(\sqrt{1.057972\frac{E_{s}}{N_{0}}}\right)+Q\left(\sqrt{1.077062\frac{E_{s}}{N_{0}}}\right)+\frac{3}{2}Q\left(\sqrt{1.090005\frac{E_{s}}{N_{0}}}\right)\\ \; & -13Q\left(\sqrt{1.080352\frac{E_{s}}{N_{0}}}\right)Q\left(\sqrt{0.360117\frac{E_{s}}{N_{0}}}\right)+10Q^{2}\left(\sqrt{0.720235\frac{E_{s}}{N_{0}}}\right)\;. \end{array}$$

For comparison purposes, the symbol error probability for orthogonal 4FSK can be well approximated by [10]

(143) $$P_{e}\approx 3Q\left(\sqrt{\frac{E_{s}}{N_{0}}}\right)-13Q\left(\sqrt{\frac{E_{s}}{N_{0}}}\right)Q\left(\sqrt{\frac{E_{s}}{3N_{0}}}\right)+10Q^{2}\left(\sqrt{\frac{2E_{s}}{3N_{0}}}\right)\;.$$

## 7. Validation

The purpose of this section is twofold. First, we want to compare the symbol error probability of the proposed optimum nonorthogonal MFSK schemes with some benchmark schemes, such as orthogonal MFSK [1] and the nonorthogonal MFSK schemes proposed by Sagias et al. in [31]. Second, we want to validate the theoretical analysis of Section 6 by comparing the theoretical symbol error probability with the simulated symbol error rate (SER).

In this section, the simulated SER is estimated by transmitting $N_{\mathrm{sym}}=10^{8}$ symbols for each simulation point, identified by a marker at the corresponding value of signal-to-noise ratio (SNR), defined as $E_{s}/N_{0}$. The $N_{\mathrm{sym}}$ symbols, independent and identically distributed, are randomly generated vectors of the *M*-dimensional signal space, as expressed by (117)–(120) for 4FSK. For each value of SNR, whose quantization step is 0.25 dB, a different set of $N_{\mathrm{sym}}$ transmitted symbols and $N_{\mathrm{sym}}$ received AWGN vectors are generated. We consider a target symbol error probability in the range $10^{-4}\leq P_{e}\leq 10^{-1}$, which is typical for uncoded systems; hence, the simulated SER is a random variable with expected value $\eta_{\mathrm{SER}}=P_{e}$ and standard deviation $\sigma_{\mathrm{SER}}=\sqrt{P_{e}\left(1-P_{e}\right)/N_{\mathrm{sym}}}\approx \sqrt{P_{e}}\cdot 10^{-4}$.

**Figure 3:**
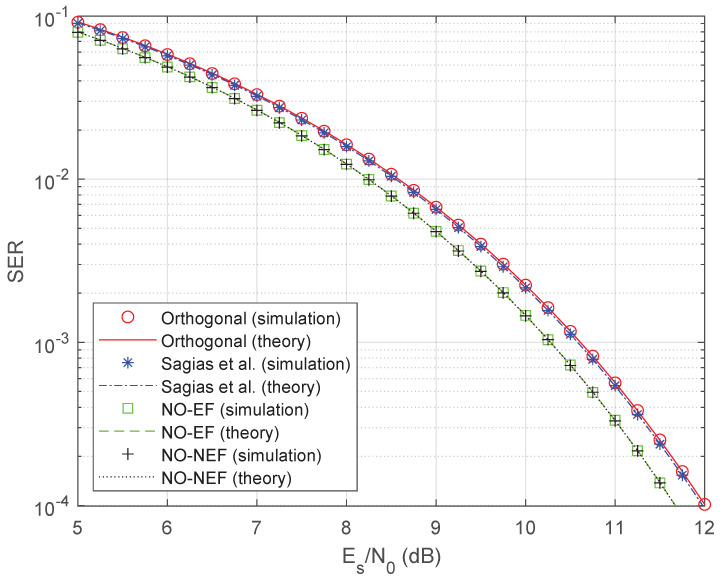
Simulated SER and theoretical symbol error probability of 4FSK schemes. The designed optimum nonorthogonal 4FSK scheme with equispaced frequencies is denoted as NO-EF, while the designed optimum nonorthogonal 4FSK scheme with non-equispaced frequencies is denoted as NO-NEF.

Figure 3 compares the symbol error probability of 4FSK schemes versus the SNR $E_{s}/N_{0}$. The theoretical symbol error probability is given by (141) for the proposed optimum nonorthogonal 4FSK with non-equispaced frequencies (denoted as NO-NEF), by (142) for the optimum nonorthogonal 4FSK with equispaced frequencies (denoted as NO-EF), and by (143) for the orthogonal 4FSK [1]. For the nonorthogonal 4FSK of Sagias et al. [31], the theoretical symbol error probability is obtained by inserting the crosscorrelation coefficients derived in [31] into (140). For all 4FSK schemes, the theoretical analysis well matches the simulated SER results. Figure 3 shows that both the proposed optimum schemes (NO-NEF and NO-EF) outperform the two benchmark schemes (orthogonal [1] and Sagias et al. [31]), thus confirming the validity of the proposed optimization. It is worth noting that the optimized 4FSK scheme with equispaced frequencies also outperforms the 4FSK scheme with non-equispaced frequencies proposed in [31]. The reason why our proposed 4FSK schemes outperform the two benchmark 4FSK schemes lies in the increased minimum squared Euclidean distance between symbol waveforms, defined as $d_{\min}^{2}=\min{\left\{d_{m,n}^{2}\right\}}_{m\neq n}$, where $d_{m,n}^{2}$ is defined in (6): for the proposed schemes, the crosscorrelation coefficients are all negative, hence $d_{\min}^{2}>\sqrt{2E_{s}}$, while some crosscorrelation coefficients are nonnegative in the benchmark schemes, leading to $d_{\min}^{2}\leq \sqrt{2E_{s}}$.

**Figure 4:**
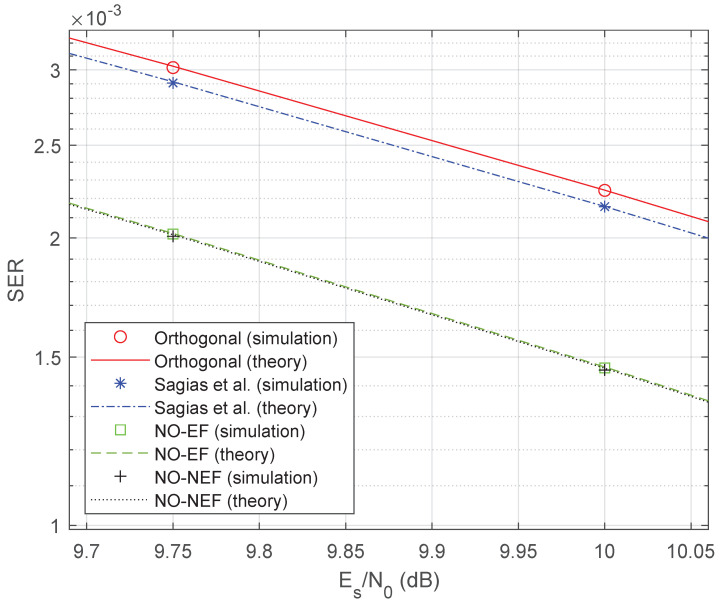
Zoom on the SER comparison of Figure 3.

To quantify the performance improvement of the proposed 4FSK schemes, Figure 4 displays a zoom around $E_{s}/N_{0}=10$ dB, using a reference SER value $P_{e}=2\cdot 10^{-3}$ similar to [31]. Figure 4 shows that the 4FSK of Sagias et al. [31] has an SNR gain of 0.03 dB only with respect to orthogonal 4FSK [1], while both proposed 4FSK schemes have an SNR gain of 0.30 dB with respect to Sagias et al., with a consequent gain of 0.33 dB with respect to orthogonal 4FSK. This 0.33 dB gain is larger than the rough estimate $10\log_{10}\left(1-\rho_{\mathrm{opt}}\right)\approx 0.24$ dB provided by the coefficient $\rho_{\mathrm{opt}}=-0.057972$ in Table 1.

**Figure 5:**
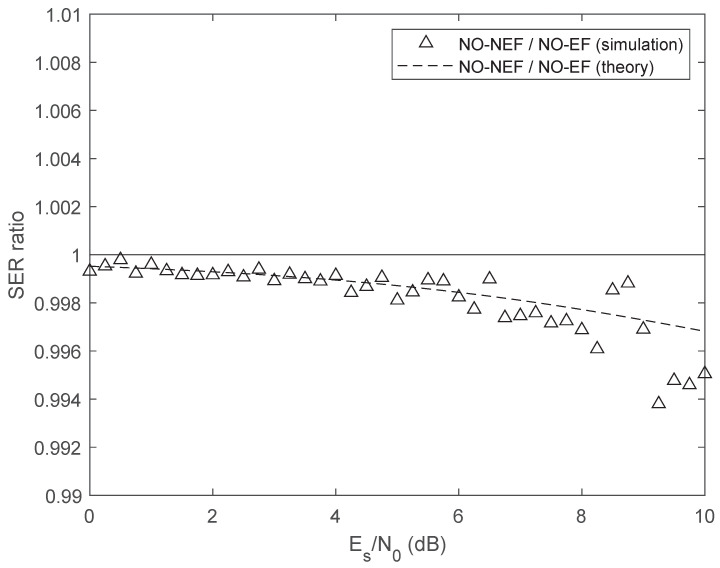
Ratio between the SER of the optimum 4FSK scheme with non-equispaced frequencies and the SER of the optimum 4FSK scheme with equispaced frequencies.

Figure 5 exhibits the ratio between the SER of the two proposed 4FSK schemes. Both the theoretical ratio and the simulated values confirm that the proposed optimal scheme NO-NEF with non-equispaced frequencies has lower SER than the optimal scheme NO-EF with equispaced frequencies. However, the SER ratio is close to one; hence, the SER difference between the two schemes is rather small. This result is the consequence of Theorem 8: according to (37), both optimized schemes share the same maximum crosscorrelation coefficient, and hence, the minimum Euclidean distance between symbol waveforms is the same for both schemes.

**Figure 6:**
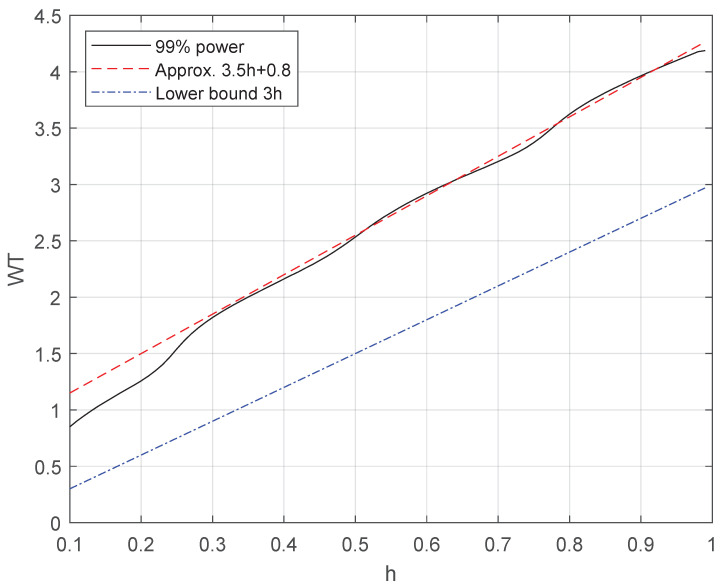
Ninety-nine percent bandwidth *W*, normalized with respect to the symbol rate 1/T, of 4FSK with equispaced frequencies, as a function of the modulation index *h*.

Figure 6 illustrates the 99% bandwidth *W* of 4FSK with equispaced frequencies, normalized with respect to the symbol interval *T*, as a function of the modulation index *h*. The 99% bandwidth *W* has been computed by numerical integration of the power spectral density of CPFSK given in [1]. Figure 6 also includes the lower bound (23) expressed by $W_{\mathrm{LB}}=f_{M}-f_{1}=3h/T$ and the bandwidth approximation

(144) $$W_{\mathrm{approx}}=\frac{3.5h+0.8}{T}\;,\;0.3\leq h<1\;,$$

derived by linear least-squares regression on the 99% bandwidth *W*. According to (144), the 99% bandwidth of orthogonal 4FSK can be approximated as $W_{\mathrm{orth}}\approx 2.55/T$, while the proposed NO-EF signal has a larger bandwidth, approximated using (144) as $W_{\mathrm{NO}-\mathrm{EF}}\approx 4/T$, i.e., a 57% bandwidth increase with respect to orthogonal 4FSK. Equation (144), derived for 4FSK with equispaced frequencies, can be exploited also for obtaining a conservative estimation of the 99% bandwidth of 4FSK with non-equispaced frequencies, provided that *h* in (144) is replaced by $\max{\left\{h_{m,m+1}\right\}}_{1\leq m\leq 3}$. Consequently, the 99% bandwidth of the proposed NO-NEF signal can be approximated as $W_{\mathrm{NO}-\mathrm{NEF}}\approx \left(3.5\max\left\{h_{m,m+1}\right\}+0.8\right)/T\approx 4.03/T$. Using the same approximation, the 99% bandwidth for the 4FSK signal of [31] can be expressed as $W_{S}\approx \left(3.5\max\left\{h_{m,m+1}\right\}+0.8\right)/T\approx 2.63/T$. Hence, the improved performance of the proposed 4FSK schemes is accompanied with a larger bandwidth: the percentage increase is approximately 52–53% with respect to the 4FSK of [31].

**Figure 7:**
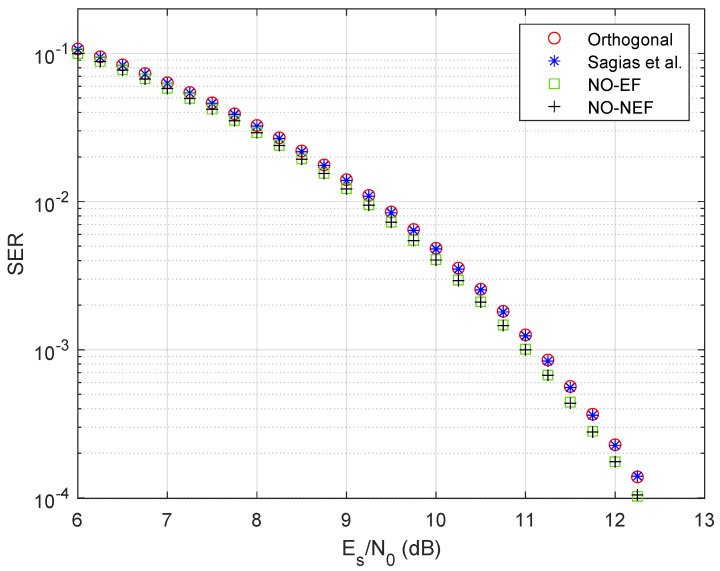
Simulated SER of 8FSK schemes. NO-EF stands for the designed optimum 8FSK scheme with equispaced frequencies, while NO-NEF stands for the designed optimum 8FSK scheme with non-equispaced frequencies.

**Figure 8:**
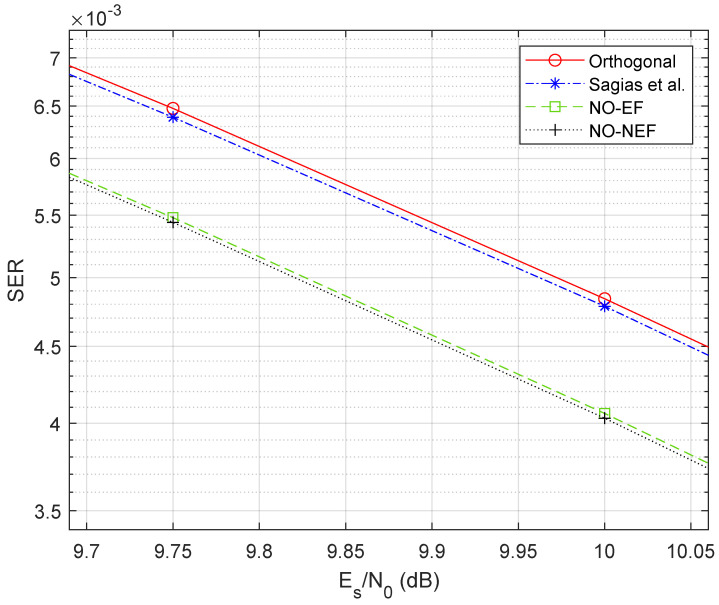
Zoomed view on the SER of Figure 7, with added lines for improved readability.

**Figure 9:**
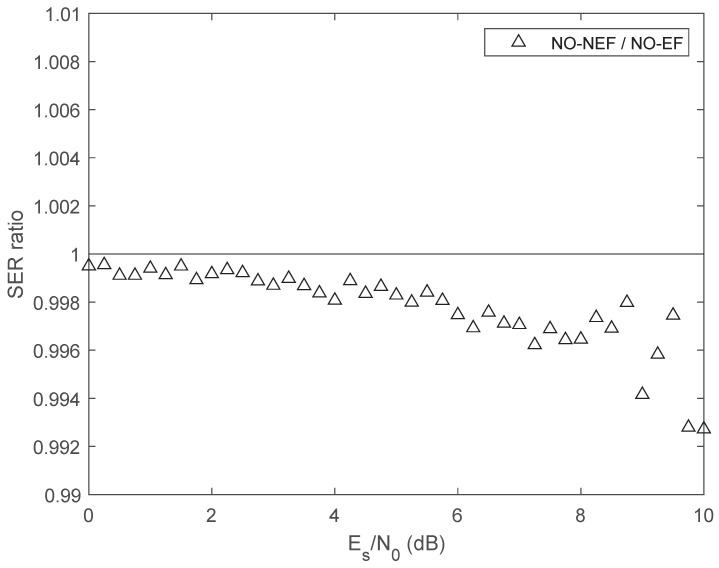
Ratio between the SER of the optimum 8FSK scheme with non-equispaced frequencies and the SER of the optimum 8FSK scheme with equispaced frequencies.

The performance results obtained for 8FSK fully confirm the corresponding results previously discussed for 4FSK. Figure 7 compares the simulated SER of the proposed nonorthogonal 8FSK schemes with orthogonal 8FSK and with the nonorthogonal 8FSK of Sagias et al. [31]. Similarly to 4FSK in Figure 3, both the proposed optimum 8FSK schemes (NO-NEF and NO-EF) outperform the two benchmark 8FSK schemes. This is a further corroboration of the validity of our optimization. As highlighted in Figure 8, which zooms the performance around $E_{s}/N_{0}=10$ dB and SER $P_{e}=5\cdot 10^{-3}$ like in [31], the 8FSK of Sagias et al. [31] has a low SNR gain (around 0.01 dB) with respect to orthogonal 8FSK, while the proposed 8FSK scheme denoted as NO-NEF has an SNR gain of 0.15 dB with respect to orthogonal 8FSK. Again, the 0.15 dB gain is larger than the rough estimate $10\log_{10}\left(1-\rho_{\mathrm{opt}}\right)\approx 0.10$ dB, with $\rho_{\mathrm{opt}}=-0.023585$ from Table 1. Also in the 8FSK case, the proposed NO-NEF scheme slightly outperforms the proposed NO-EF scheme, with a difference of about 0.01 dB. This small difference is underlined by Figure 9, which presents the ratio between the SER of the two proposed 8FSK schemes. In addition, the proposed 8FSK scheme with equispaced frequencies, denoted as NO-EF, outperforms the 8FSK scheme with non-equispaced frequencies proposed in [31], with a gain of 0.13 dB. This confirms that the designs of [31] are suboptimal, from the performance viewpoint. Similarly to 4FSK, in 8FSK, the price paid for our performance gain is also a larger bandwidth, caused by using a modulation index h=0.963749 close to one, while the normalized frequency separations in [31] are close to h=0.5 used by orthogonal 8FSK.

## 8. Conclusions

This paper has proposed a performance optimization of nonorthogonal MFSK with symbol-by-symbol coherent detection. The first part of this paper has analytically derived the mathematical conditions for the design of MFSK waveforms with negative crosscorrelation between symbol waveforms. Differently from the existing literature, our approach proves that the Euclidean distance between the two closest symbol waveforms is larger than $\sqrt{2E_{s}}$, thereby reducing the errors caused by noise.

The second part of this paper has analytically optimized the performance of nonorthogonal MFSK with equispaced frequencies. Specifically, we have mathematically determined the optimum modulation index that maximizes the minimum distance among the symbol waveforms. Differently from the existing literature, where the MFSK design is mainly based on computer-based numerical search, our theoretical approach builds on the mathematical conditions derived in the first part of this paper. As a result of our theoretical optimization, our MFSK design with equispaced frequencies outperforms existing MFSK designs based on numerical optimization, like [31].

The third part of this paper has analytically optimized the performance of nonorthogonal MFSK with non-equispaced frequencies, determining, for both 4FSK and 8FSK, the optimum frequency separations that maximize the minimum distance among the symbol waveforms. Differently from the existing literature, we have proved that the use of non-equispaced frequencies cannot increase the minimum Euclidean distance between the two closest symbol waveforms, with respect the optimum MFSK with equispaced frequencies. Therefore, the additional performance gain given by non-equispaced frequencies is limited and comes from the maximization of the Euclidean distance between the second closest symbol waveforms. This performance gain has been quantified (analytically for 4FSK and with simulations for 8FSK), highlighting the amount of performance improvement with respect to existing MFSK schemes. This paper does not omit the limitations of the proposed MFSK schemes, such as the additional bandwidth requirements with respect to orthogonal MFSK. This increased bandwidth is the price to pay for the performance improvement provided by the proposed schemes.

In our study, we have chosen to ignore other types of channels, such as fading channels. The motivation for this choice is explained in the following. The symbol error probability in fading channels is usually obtained by integrating the symbol error probability in AWGN over the probability density function of the fading coefficient [1,34,35]. Since the designed MFSK schemes have reduced symbol error probability for all the SNR values, with respect to the benchmark schemes, it is straightforward that the designed MFSK schemes surely outperform the benchmark schemes in fading channels. The derivation of the symbol error probability in fading channels, for some selected nonorthogonal MFSK schemes and fading statistics, could be the subject of future work.

## Figures and Tables

**Table 1:** Optimum modulation index $h_{\mathrm{opt}}$ and its approximation $h_{\mathrm{approx}}$.

*M*	$h_{\mathrm{opt}}$	$h_{\mathrm{approx}}$	$\rho_{\mathrm{opt}}$	$\rho_{\mathrm{approx}}$
2	0.715148	0.750000	−0.217234	−0.212207
3	0.867722	0.875000	−0.091325	−0.090946
4	0.913589	0.916667	−0.057972	−0.057875
5	0.935809	0.937500	−0.042480	−0.042441
6	0.948933	0.950000	−0.033525	−0.033506
7	0.957599	0.958333	−0.027690	−0.027679
8	0.963749	0.964286	−0.023585	−0.023579
16	0.983219	0.983333	−0.010791	−0.010790
32	0.991909	0.991935	−0.005176	−0.005176
64	0.996025	0.996032	−0.002536	−0.002536
128	0.998030	0.998031	−0.001256	−0.001256
256	0.999019	0.999020	−0.000625	−0.000625

## Data Availability

The data used in this study are either contained in the paper body or randomly generated as detailed in the paper body. No external datasets have been used in this study.

## Abbreviations

The following abbreviations are used in this manuscript:

ANCC	All-negative crosscorrelation coefficients
AWGN	Additive white Gaussian noise
CPFSK	Continuous phase frequency-shift keying
CPM	Continuous phase modulation
FSK	Frequency-shift keying
IoT	Internet of Things
ISAC	Integrated sensing and communication
MFSK	M-ary frequency-shift keying
NO-EF	Nonorthogonal with equispaced frequencies
NO-NEF	Nonorthogonal with non-equispaced frequencies
PLC	Powerline communication
QAM	Quadrature-amplitude modulation
SER	Symbol error rate
SNR	Signal-to-noise ratio
SWIPT	Simultaneous wireless information and power transfer
UWAC	Underwater acoustic communication
VLC	Visible light communication
